# Population Genetics Meets Ecology: A Guide to Individual‐Based Simulations in Continuous Landscapes

**DOI:** 10.1002/ece3.71098

**Published:** 2025-04-15

**Authors:** Elizabeth T. Chevy, Jiseon Min, Victoria Caudill, Samuel E. Champer, Benjamin C. Haller, Clara T. Rehmann, Chris C. R. Smith, Silas Tittes, Philipp W. Messer, Andrew D. Kern, Sohini Ramachandran, Peter L. Ralph

**Affiliations:** ^1^ Center for Computational Molecular Biology Brown University Providence Rhode Island USA; ^2^ Institute of Ecology and Evolution University of Oregon Eugene Oregon USA; ^3^ Department of Computational Biology Cornell University Ithaca New York USA; ^4^ Department of Biology University of Oregon Eugene Oregon USA; ^5^ Department of Data Science University of Oregon Eugene Oregon USA

## Abstract

Individual‐based simulation has become an increasingly crucial tool for many fields of population biology. However, continuous geography is important to many applications, and implementing realistic and stable simulations in continuous space presents a variety of difficulties, from modeling choices to computational efficiency. This paper aims to be a practical guide to spatial simulation, helping researchers to implement individual‐based simulations and avoid common pitfalls. To do this, we delve into mechanisms of mating, reproduction, density‐dependent feedback, and dispersal, all of which may vary across the landscape, discuss how these affect population dynamics, and describe how to parameterize simulations in convenient ways (for instance, to achieve a desired population density). We also demonstrate how to implement these models using the current version of the individual‐based simulator, SLiM. We additionally discuss natural selection—in particular, how genetic variation can affect demographic processes. Finally, we provide four short vignettes: simulations of pikas that shift their range up a mountain as temperatures rise; mosquitoes that live in rivers as juveniles and experience seasonally changing habitat; cane toads that expand across Australia, reaching 120 million individuals; and monarch butterflies whose populations are regulated by an explicitly modeled resource (milkweed).

## Graphical abstract

This paper aims to be a practical guide to spatial simulation, helping researchers to implement realistic and efficient spatial, individual‐based simulations and avoid common pitfalls. We explore how mating, reproduction, density‐dependent feedback, dispersal, spatial and temporal heterogeneity, and natural selection affect population dynamics and present guides on how to build effective simulations on the current version of SLiM.

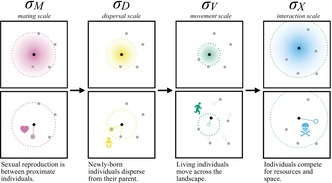

## 1 Introduction

Explicit spatial models are indispensable for understanding how species live, interact, and evolve across geographic landscapes. However, formulating sensible models of demography in continuous space is fraught with pitfalls and choices unfamiliar to many researchers interested in spatial modeling. For instance, in the commonly used, nonspatial Wright–Fisher model, the population size is directly specified. However, in spatial models with locally defined dynamics, the number of individuals is a stochastic, emergent property. It takes some expertise to coerce the model to produce a desired equilibrium size. Even fundamental population‐genetic concepts such as selection coefficients cease to have a single obvious interpretation in a spatial context.

Implementing an individual‐based simulation requires great specificity—choices must be made regarding many mechanisms and parameters. Simulated organisms must separately give birth and die, unlike in more abstract theory where often only the net effects of birth minus death enter (e.g., Cantrell and Cosner 2004). Here we present modeling strategies for implementing individual‐based simulations in explicit geographic space. These include spatial movement, such as dispersal, as well as spatial interactions, such as the feedback between local population density and net reproductive rate that is necessary to avoid unbounded growth.

Why individual‐based simulations, and why in continuous space? Since real individuals are discrete, and live in continuous space, such simulations can in principle more accurately model many real‐world situations. Some phenomena simply require individual‐based simulations (e.g., locally adaptive genotypes). Non‐individual‐based models can be more computationally efficient, but at an often unknown cost to accuracy (Stillman et al. 2015). Similarly, discretized spatial landscapes are usually associated with model assumptions that do not provide a consistent approximation to continuous‐space dynamics (e.g., Barton et al. 2002). For instance, Battey et al. (2020) showed that some aspects of genetic variation in fine grids of randomly mating demes of fixed size were irreconcilably different from continuous‐space models: in fact, discretization error increased with finer grids. Given these effects of discretization, it is in our experience simpler to move directly to continuous space. Indeed, individual‐based simulations in continuous space may even require *less* modeling expertise than other strategies, because it is usually relatively easy to come up with order‐of‐magnitude estimates of aspects of an organism's life cycle from natural history data, which can be used to parameterize an individual‐based simulation. More abstracted modeling frameworks can involve analytical approximations, compound parameters, and other technicalities that require careful checking and mathematical experience. For instance, it is more difficult to translate “offspring disperse around 100 m” to a migration rate between adjacent grid cells than to directly input a mean dispersal parameter of 100 m. Although abstract models with fewer choices might feel more general for theoretical work or methods development, this can be a false generality, as more work is required after the fact to determine to which real‐world organisms a given result or method is applicable.

Explicit individual‐based population models are not new to ecology (DeAngelis and Yurek 2017; Grimm 1999). A great deal of ecological work has sought to quantify the effects of density‐dependent demographic feedback. For a single species, negative feedback between population density and growth rate is necessary to avoid the population growing without bound, although there are a great many ways to set this up in practice (De Wit 1960; Beverton and Holt 1957; Ellner et al. 2016). Demographers have a deep understanding of how to describe and parameterize the statistical properties of birth and death, and what the emergent consequences are for population growth, lifespan, age distribution, and long‐term fitness (Tuljapurkar 2013). Although temporal stochasticity is relatively well understood in demography (Tuljapurkar 1989), the consequences of spatial heterogeneity—particularly outside of metapopulation models (Hanski 1997)—have received less attention.

Geography can have strong effects on patterns of genetic variation (Wright 1943; Malécot 1969; Rousset 2000; Charlesworth et al. 2003; Battey et al. 2020; Min et al. 2022) and on evolutionary processes (Felsenstein 1976; Uecker et al. 2014; Savolainen et al. 2007). Genetic differentiation is shaped by the movement of individuals, and hence distance, geographical features, and the spatio‐temporal history of the species (Hewitt 2011; Rosenberg et al. 2005; Ramachandran et al. 2005). Geography is, therefore, not only important, but also a relatively untapped source of information to inform inference (Bradburd and Ralph 2019). However, it is difficult to obtain analytical predictions from spatial population genetics models (Felsenstein 1975; Barton et al. 2002). Most spatial work in population genetics uses partial differential equations that do not represent genetic differentiation (Montgomery 1973; Barton 1979; Sedghifar et al. 2016; Etheridge et al. 2024), or specifically looks at the fronts of population expansions (e.g., Barton et al. 2013; Paulose et al. 2019; Nullmeier and Hallatschek 2013; Etheridge and Penington 2022).

Simulation has long been a useful tool in the study of populations (Grimm 1999; DeAngelis and Yurek 2017), particularly for the purposes of prediction (e.g., for population viability analysis, see Dunning Jr. et al. 1995)—even predating the common usage of digital computers (Pearson 1960). Simulations are also useful for inference, ranging from exploratory studies to training for deep learning. Their use depends on their computational cost: many modern machine‐learning methods are only feasible with relatively fast simulations. Introducing geographic space increases computational complexity, making the task of producing training data more difficult (but see Smith et al. 2023; Champer et al. 2021). Today, there are several sophisticated software suites targeted at just the sort of landscape‐scale simulations we discuss here, with both discretized spatial models (Landguth et al. 2017; Bocedi et al. 2021; Landguth and Cushman 2010; Schumaker and Brookes 2018; Neuenschwander et al. 2018; Rebaudo et al. 2013) and continuous spatial coordinates (Haller and Messer 2023).

### 1.1 How to Use This Paper

This paper is intended as a guide to the territory of spatial modeling, with a focus on individual‐based simulations in discrete time in continuous geographic space.

The paper has two broad parts: The first part (Sections 2 through 8) describes modeling strategies to capture ecological and geographic processes in individual‐based models. The second part, Case Studies, provides concrete examples of how to combine these modeling strategies to simulate a biological system.

First, we tackle population regulation (Section 2), as this is the topic that in our experience is the greatest barrier to new modelers. We then describe our strategy for parameterizing and analyzing spatial simulations (Section 3). Next, we cover spatial movement and mate choice (Sections 4 and 5, respectively), highlighting some interesting challenges. Subsequently, we discuss how to implement spatial heterogeneity and further concepts in population regulation, including stochasticity (Section 6), and finally natural selection (Section 8).

We hope this material will be useful to researchers who wish to produce simulations that are—at least roughly—modeled on concrete empirical systems. This includes empirical researchers wanting to explore the plausibility of hypotheses or the power of study designs, methods developers who want to test their methods on realistic spatial models, theoreticians wishing to explore spatial models, and managers wishing to explore alternative scenarios. So, we aim to make it easy to build a model starting from those quantities that we generally have good estimates of for particular organisms. This differs from the modeling philosophy in much theoretical work, which often begins from a “simplest possible” model.

To give practitioners a head start on simulating the models we describe, the main text is accompanied by Boxes containing code that implements each modeling concept. The code may be run with the program SLiM (Haller and Messer 2023), a flexible and powerful individual‐based eco‐evolutionary simulator that, as of the newly released version 4.2, includes a full set of tools for modeling interactions between multiple species across geographic landscapes. We recommend that readers open the minimal SLiM template (provided in Appendix A and at https://github.com/kr‐colab/spatial_sims_standard) in SLiM's GUI to experiment with while reading. The online code repository also contains SLiM scripts for each of the Case Studies.

## 2 Population Dynamics and Density

We start by describing how to maintain a stable population. Consider an extremely simple spatial simulation: organisms are asexual, and do not move during their lifespan. Each time step, each adult gives birth to a Poisson(f) number of offspring, that each disperse to a random location, whose displacement from their parent's location is drawn from a Normal distribution with mean 0 and standard deviation $\sigma_{D}$. Then, each individual dies with a probability μ.

Simulating this model, its flaw is immediately apparent as it runs: either all individuals rapidly disappear (if f≤μ), or the computer grinds to a halt as the number of individuals explodes (if f>μ). We need some kind of *density‐dependent feedback* to maintain a stable population size—in other words, we need the net population growth rate to change from positive to negative as the population density grows past some point. When population density in an area is high, either birth rates need to decrease or death rates need to increase.

To provide density‐dependent feedback, we first need a notion of “population density” at a point in space, a measure of the number of individuals nearby per unit area. Let K denote the desired equilibrium population density (in individuals per unit area). A general way to define $n\left(x\right)$, the population density around a location x, is to specify an *interaction kernel*, $\rho \left(x\right)$, which is a nonnegative function with $\int \rho \left(x\right)dx=1$; an *interaction scale* $\sigma_{X}$; and then if the two‐dimensional locations of the individuals in the population are $x_{1},\ldots ,x_{n}$, define $n\left(x\right)$ by

(1) $$n\left(x\right)=\frac{1}{\sigma_{X}^{2}}\sum_{i}\rho \left(\frac{x-x_{i}}{\sigma_{X}}\right).$$

Since $\int \rho \left(x/\sigma_{X}\right)dx=\sigma_{X}^{2}$, the value of $n\left(x\right)$ is in units of individuals per unit area. A common choice for $\rho \left(x\right)$ is the Gaussian density function. One concrete interpretation is that if $\rho \left(\left(x-y\right)/\sigma_{X}\right)$ gives the proportion of time that an individual at y spends near x, then $n\left(x\right)$ is proportional to the time spent by *all* individuals near x. (More concretely, $\int_{A}n\left(x\right)dx$ is the total amount of time spent by all individuals in the region A).

Now suppose that in each time step of the model, each individual has a chance to produce offspring. The number of offspring depends on the individual's location. An individual at x produces a random number of offspring with a mean of $f\left(n\left(x\right)/K\right),$ where f is the *birth rate* or *fecundity*. Each juvenile then disperses to a nearby location whose displacement from x is chosen from a given probability distribution. Then, all individuals (including those just born) survive to the next time step with probability $1-\mu \left(n\left(x\right)/K\right)$, where $\mu \left(u\right)$ is the *mortality rate* at scaled density u. This way, birth and death rates depend on an individual's location x via the smoothed population density $n\left(x\right)$, scaled by a parameter K that controls the equilibrium density. We build on this general form throughout the paper to describe how life history, geography, and selection make individual fecundity and mortality rates more complex.

### 2.1 Equilibrium Population Density

Will this model stabilize, and if so, to what density? We expect an equilibrium when births balance deaths. If we define the local *per‐capita* net reproductive rate (the expected increase due to birth minus the decrease due to death, per individual) to be

(2) $$F\left(u\right)=f\left(u\right)\left(1-\mu \left(u\right)\right)-\mu \left(u\right).$$

then we expect an equilibrium at a density of $n_{*}$, solving $F\left(n_{*}/K\right)=0$. Note that newborns are subjected to the same mortality rate $\mu \left(u\right)$ as the rest of the population, so the expected increase is $f\left(u\right)\left(1-\mu \left(u\right)\right)$ rather than $f\left(u\right)$ in Equation (2). For this reason, it is convenient to choose the functional forms of f and μ so that $F\left(1\right)=0$, in which case we expect the equilibrium $n_{*}$ to be roughly equal to K. (However, we will see in Sections 6, 7.3, and Appendix B.4 that often K is not *exactly* the equilibrium density.) In addition to $F\left(1\right)=0$, for the population to be stable we also need $F^{\prime }\left(1\right)<0$ so that the net reproductive rate decreases with density near to the equilibrium. The argument u in $f\left(u\right)$, $\mu \left(u\right)$, and $F\left(u\right)$ is the *scaled* population density $u=n\left(x\right)/K$, written this way so that it is easy to control the equilibrium population density by simply changing K, independently of other factors.

A brief note on what we have done here: it might seem most natural to set up a simulation using fecundity and mortality rates based on empirical observation. If so, then mean population density would be an emergent property of the simulation—in other words, our script would not have a parameter K that we could directly adjust. However, to do this we need empirical estimates of how fecundity and mortality depend on local density, which are very difficult to obtain. A much more common situation is to have estimates of fecundity and mortality rates *and population density* at equilibrium. The parameterization we outline here, in which K is a directly tunable rather than emergent parameter, is designed to make this use case natural.

#### 2.1.1 Functional Forms

The next question is: what forms should we use for the birth and death rate functions $f\left(u\right)$ and $\mu \left(u\right)$? There is surprisingly little guidance from the theoretical or empirical literature. Population models mostly come in two flavors: “phenomenological” (or “top‐down”) models that only consider the net reproductive rate, $F\left(u\right)$; and “mechanistic” (or “bottom‐up”) models that explicitly consider birth and death separately (Geritz and Kisdi 2012). We need a mechanistic model, since our approach here is individual‐based, but most literature on density dependence uses phenomenological models (for notable exceptions, see Coulson et al. (2008), or integral projection models Ellner et al. (2016)). Matrix population models (Caswell 2000) are (mostly) mechanistic and widely used for management, but rarely incorporate density dependence. Estimating the functional forms of density dependence from empirical data is a logistically and statistically daunting task, even without considering environmental variation. As we intend to simulate from our model, we will simply choose a mathematically convenient form, reducing the problem to estimating parameters given that functional form. Perhaps the most obvious strategy is to multiply the base fecundity or survival rate by a function of the local density that decreases when density is high. Appendix C works through examples using common functional forms for $F\left(u\right)$.

### 2.2 Regulation by Mortality

Suppose we would like to use density‐dependent feedback only on mortality. In this case, the average number of offspring is constant: we may say $f\left(u\right)=f$. Rearranging Equation (2), we see the survival probability in a location experiencing a scaled population density u is

(3) $$1-\mu \left(u\right)=\frac{1+F\left(u\right)}{1+f}.$$

The Beverton–Holt form for the net effects of density (Beverton and Holt 1957) would have that $F\left(u\right)$ is proportional to $\left(1+a\right)/\left(1+\mathrm{au}\right)-1$, for some constant a that controls the strength of the density‐dependent feedback. So, to set up the model to have “Beverton–Holt” feedback, we plug in $F\left(u\right)=\left(1+a\right)/\left(1+\mathrm{au}\right)-1$ and obtain that

(4) $$1-\mu \left(u\right)=\frac{1+a}{\left(1+f\right)\left(1+\mathrm{au}\right)}\;\text{if}\;a\leq f.$$

**FIGURE 1:**
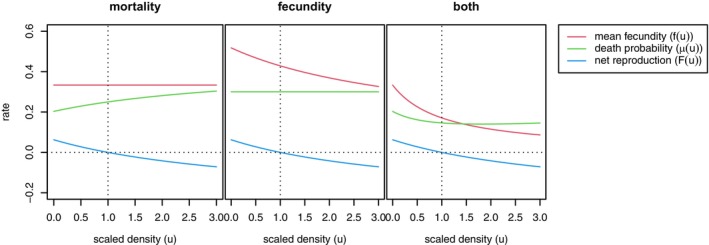
Three example models with “Beverton–Holt” regulation of population density. The relationship between scaled local population density and vital rates: Mean fecundity $f\left(u\right)$, probability of death $\mu \left(u\right)$, and net reproduction rate $F\left(u\right)=f\left(u\right)-\mu \left(u\right)$. In each, the horizontal axis is shown in units of K, the parameter controlling equilibrium density (“carrying capacity”). The models are mortality from Equation (4), fecundity from Equation (5), and both from Equation (6).

The leftmost plot in Figure 1 visualizes Equations (3) and (4); the others are discussed in Section 7. Mortality regulation is also demonstrated in Box 1, and most of the examples in this paper use this model with a=f. In practice, an empirical estimate of the survival probability at low density (the limit as u→0) $s_{0}$ can be incorporated so that $a=s_{0}\left(1+f\right)-1$.

The condition that a≤f is so that this gives us a valid probability: otherwise, the survival probability can be negative. Note that the strategy used here cannot work with the discrete logistic: since $F\left(u\right)=1-u$ can get arbitrarily negative, eventually random density fluctuations will lead to negative probabilities, and errors in the simulation.

There are many other possible ways to set up $f\left(u\right)$ and $\mu \left(u\right)$ that result in the same net form of density dependence. (See Section 7.3 and Appendix C for how stochasticity alters the realized equilibrium density and alternative functional forms.) The general forms of $f\left(u\right)$ and $\mu \left(u\right)$ may also need to be modified depending on the organism. If there are two sexes and only one sex can bear offspring, then f should not be the total number of offspring, but rather the number of offspring *of the offspring‐bearing sex*. More generally, if vital rates depend on any aspect of the individual (e.g., only adults can reproduce, or mortality is age‐dependent), then the equivalent calculations must be done with a matrix population model (Caswell 2000) or an integral projection model (Ellner et al. 2016). Our examples mostly ignore such complications, although several models have distinct life stages (Case Studies 9.2 and 9.4).

BOX 1 **Regulating mortality and fecundity in SLiM.**

The Wright–Fisher model is a population model with a *fixed* population size. A Wright–Fisher model in continuous space, therefore, has *global* population regulation—individuals are affected by others arbitrarily far away, resulting in counter‐intuitive, unrealistic consequences (Felsenstein 1975). SLiM's default model is the Wright–Fisher model (“WF”). In this study, our goal is to model particular species in realistic ways, so we use only SLiM's non‐Wright–Fisher (“nonWF”) model, which requires explicitly choosing how births and deaths occur.

In a SLiM nonWF model, the “fitness” attribute of an individual is the probability of survival until the next time step. So, controlling mortality in SLiM is simply a matter of setting individuals' fitness. To compute local density, we use the localPopulationDensity() function, which computes density just as described in Equation (1) (with options for the choice of kernel). To do this we need to first (during setup) define an InteractionType object, here using a Normal kernel with a scale of SX (as in, $\sigma_{X}$) and a maximum distance of (SX * 3):

1	initializeInteractionType(1, “xy”, maxDistance=SX * 3);2	i1.setInteractionFunction(“n”, 1.0, SX);

Now, to use Beverton–Holt regulation on mortality as described around Equation (4) (setting a=1 in Equation (4) and with K defined elsewhere), in each time step, we use this interaction type to set survival probabilities:

3	inds = p1.individuals;4	density = i1.localPopulationDensity(inds);5	u = density / ((1 + f) * K);6	inds.fitnessScaling = 1 / (1 + f * u);

Note that the local population density is measured after reproduction and before death ($N_{t}^{+}$ in Appendix B), so we divide by 1+f as well as K when converting it to scaled population density u from the Equation (1), so it reflects density *before* reproduction.

On the other hand, offspring are produced by a “reproduction() callback,” a chunk of code that is executed each time step for each individual, and produces any desired new offspring for the focal individual. For instance, if we would like to use Ricker regulation on fecundity (see Equation (19) with α=1 and β=0), we might for efficiency pre‐compute each individual's number of offspring:

7	inds.tag = rpois(length(inds), lambda=exp(-density/K));

and then in the reproduction() callback produce the offspring:

8	p1.addCrossed(individual, mate, count=individual.tag);

## 3 Spatial Scales and Neighborhood Sizes

**FIGURE 2:**
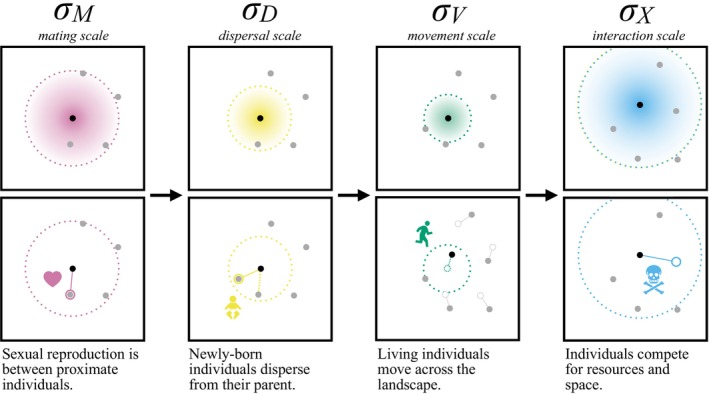
Spatial processes act over spatial scales defined by the σ parameters (radii of dotted circles). In one simulation time step, mate choice, offspring dispersal, adult movement and interindividual interaction (columns) all take place. (The order of these events is shown here as performed in SLiM.) The scale over which these processes occur are parameterized by $\sigma_{M}$, $\sigma_{D}$, $\sigma_{V}$, and $\sigma_{X}$, respectively; they can be used to calculate neighborhood sizes, to define the scale of the kernels used to draw movement or dispersal vectors (see Section 4) and to estimate local population density (see Section 5).

Now that we understand how to obtain stable simulations, we can move on to a more spatial topic: that of spatial scale. It is convenient to use spatial scale parameters to describe distance within the model; some are depicted in Figure 2: (i) interaction scale $\sigma_{X}$, the typical distance over which individuals affect each other ecologically, (ii) dispersal scale $\sigma_{D}$, the typical distance between parent and offspring, (iii) mate choice scale, $\sigma_{M}$, the typical distance between mates, and (iv) movement scale $\sigma_{V}$, the typical displacement of an individual each time step. ($\sigma_{D}$ is often called “dispersal distance,” but here we use “dispersal scale” for consistency.) We met $\sigma_{X}$ in Equation (1), where it determined which individuals were close enough to each other to affect local density.

Each spatial scale parameter determines how many other individuals typically exist in an individual's “neighborhood.” First, the *mating neighborhood size* $N_{M}=4{\pi \sigma }_{M}^{2}K$ and the *interaction neighborhood size* $N_{X}=4{\pi \sigma }_{X}^{2}K$, measure, respectively, the typical number of other individuals in a circle of radius $2\sigma_{M}$ or $2\sigma_{X}$ at equilibrium.

In practice, these measures are only intended to be order‐of‐magnitude diagnostics. For example, $N_{M}$ should be the same order of magnitude as the number of potential mates; if it is small then mate limitation may be a problem. That said, depending on the mating kernel (i.e., shape of the distribution that determines how “attractive” nearby mates are), individuals as far away as $3\sigma_{M}$ are probably also available to mate (See Section 5).

Less obvious but equally important is $N_{X}$, which measures the typical number of other individuals that “count toward” the local density of a given individual, and hence affect its demographic rates (such as survival probability, as in Box 1). $N_{X}$ also provides a measure of individual‐to‐individual variability in local density. Roughly speaking, a larger $N_{X}$ means that local density is obtained by averaging over an area with more individuals, meaning that individuals across the landscape experience more similar local densities. When $N_{X}$ is small, densities across the landscape can be more noisy—with some individuals experiencing no competition from neighbors, and some experiencing high density. Further discussion of how $N_{X}$ and $N_{M}$ can help diagnose odd model behavior is given in Appendices B.2 and B.3.

Another “neighborhood size” is a classical one: Wright's neighborhood size $N_{W}=4{\pi \sigma }_{\mathrm{eff}}^{2}K$, where $\sigma_{\mathrm{eff}}^{2}$ is the (squared) *effective dispersal distance*, the variance of the displacement between parent and offspring along any axis looking back along a lineage (chain of parent–offspring relationships). This quantity appears frequently in work on continuous spatial models in population genetics (e.g., Wright 1943, 1946; Barton et al. 2002; Rousset 1997; Robledo‐Arnuncio and Rousset 2010), and is clearly affected by $\sigma_{D}$, $\sigma_{V}$, and $\sigma_{M}$, as well as the mean generation time if adults move, but no explicit expression for $\sigma_{\mathrm{eff}}$ from these parameters is known. $N_{W}$ gives, roughly, the number of “potential parents” of a given individual, and so is a measure of the rate of local genetic drift. If $N_{W}$ is small, then local inbreeding (and spatial structure more generally) will be stronger.

Although we encourage basing modeling decisions on empirical understanding, it is not always feasible. For instance, suppose one is simulating a species of fairly common shrub that lives widely across a landscape. In practice, its local density is determined by microhabitat and complex interactions with other species. Any concrete estimate of the interaction scale for a plant is probably quite small—the competitive effect of one shrub on another more than a few meters away is (in the short term at least) usually quite small. However, implementing a spatial model with an interaction scale of only a few meters (and no other species) will likely lead to a population size that is much too large. One option is to somehow include other species and fine‐scale habitat suitability, but doing this in a realistic and efficient way can be a major challenge. A simpler option is to set the interaction scale to be on the order of the mean interindividual spacing and adjust the form of density dependence to roughly match the observed population density. The simulated population will probably be more evenly spread out across space than in reality, but it is hopefully at least a better approximation than a nonspatial model. More work is needed to develop appropriate modeling strategies for such situations and to understand their consequences.

BOX 2 **Summarizing the state of the population.**

SLiM's GUI lets the user visualize the state of the simulation as it unfolds. We can customize the display to see areas of higher fecundity, differences in age structure, or even local adaptation.

One strategy is to set the color of each individual: by default, individuals are colored by survival probability, but this code snippet will set the color to reflect the sex of each individual:

9	females = p1.subsetIndividuals(sex = “F”);10	females.color = “white”;11	males = p1.subsetIndividuals(sex = “M”);12	males.color = “red”;

Another strategy is to summarize the state of the population as a map. This can be done with the summarizeIndividuals() function, which creates a rasterized map for which the value of each pixel is some summary of the individuals within that pixel.

For instance, the map of density shown as the background was made using the following code:

13	density = summarizeIndividuals(p1.individuals, c(25, 25), p1.spatialBounds,14		operation=”individuals.size();”,15		empty=0.0, perUnitArea=T);16	p1.defineSpatialMap(“density”, “xy”, density, T, c(0,K), colors(20, “viridis”));

In this close‐up screenshot of a dioecious simulation, there tend to be more females (white points) where the local density is higher (yellow background); and there tend to be more males (red points) where the density is lower (blue background). We discuss the heterogeneous spatial patterns generated in dioecious simulations in Section 5. See Box 5 for more use of defineSpatialMap().

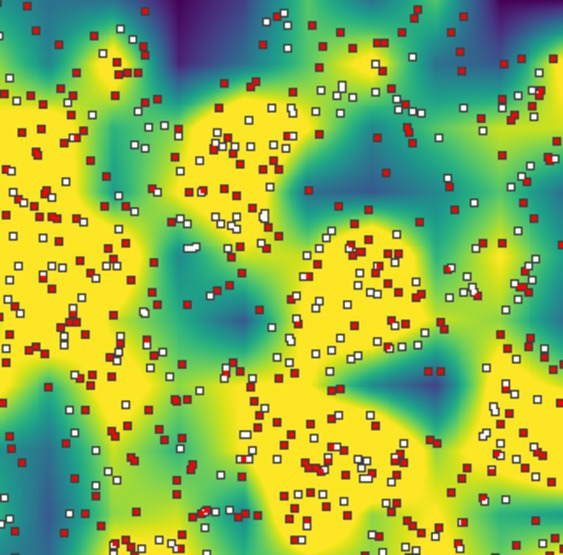

## 4 Movement and Dispersal

How individuals move across the landscape influences both their spatial distribution and how related they will be to nearby individuals. We consider parent–offspring dispersal and the movement of organisms during their lifetimes as different processes, but they can be implemented in similar ways. To model dispersal and movement, we need to consider both the overall scales of movement ($\sigma_{D}$ and $\sigma_{V}$; introduced in Section 3), as well as the shape of the dispersal distribution, or *kernel*.

The easiest way to implement dispersal in two dimensions is simply to say that an individual at location $x=\left(x,y\right)$ will produce an offspring that lives at $\left(x+\sigma_{D}X,y+\sigma_{D}Y\right)$, where X and Y are independent draws from a Gaussian. Most simulations in this paper were done in this way, for familiarity rather than any particular reason.

BOX 3 **Random dispersal and displacement.**

Perturbing a spatial location by adding a displacement drawn from a given kernel is a common operation in simulations—for instance, to choose offspring locations. To do this, SLiM provides the pointDeviated() method, which also needs to know the shape of the kernel, the type of boundary condition, and other parameters. For instance, the following code:

17	locs = subpop.pointDeviated(nOff, individual.spatialPosition, “reprising”,18			INF, “t”, DF, SD);19	offsprings = subpop.addCrossed(individual, mate, count=nOff);20	offsprings.setSpatialPosition(locs);

creates nOff offspring, and sets each offspring's position to a randomly sampled location near the location of the parent (individual). The random displacement is drawn with density $f\left(x\right)\propto {\left(1+∥x/\mathrm{SD}{∥}^{2}/\mathrm{DF}\right)}^{-\left(\mathrm{DF}+1\right)/2}$, that is, from the kernel whose density is formed by rotating the t distribution with scale SD (as in $\sigma_{D}$) and DF degrees of freedom about the origin. (See Appendix D for other strategies.) The reprising argument conditions the result on falling within the spatial bounds of the simulation—other options include stopping and reflecting. (Or for an absorbing boundary, none, with offspring falling outside the area removed).

Although the scale of movement most strongly affects spatial patterns, the shape of the kernel is also important, and can have surprising effects: even very rare long‐range movement can have strong effects on the speed of a range expansion (Mollison 1972; Paulose et al. 2019) or the relationship between genetic and geographic distances (Smith and Weissman 2023). To see the effects of rare, long‐range movement, a convenient “fat‐tailed” kernel is the Student's t: the smaller the degrees of freedom parameter, the more likely are extremely long movements.

How to draw from a different dispersal kernel? The first guess—choose X and Y from a different distribution—does *not* work: the result will not be rotationally symmetric, and dispersal will tend to fall along the x or y directions. Options are to either move a random distance at a uniformly chosen angle (which is conceptually simpler), or to multiply a bivariate Gaussian by a random scaling factor (which has other advantages). What to call a given two‐dimensional kernel is not standard—for instance, would a “Student's t kernel” have a t‐distributed distance? Or, a t‐shaped cross‐section? In simulations below we use the latter convention, as described in Box 3, and discuss these choices more in Appendix D.

The behavior of individuals near boundaries must of course be specified. Common choices include “reflecting” and “absorbing”. These differ substantially: imagine the new individual as taking a straight‐line path from $\left(x,y\right)$ in the direction of $\left(X,Y\right)$ and encountering a boundary either bounce off (reflecting) or die (absorbing). The latter clearly reduces the effective fecundity of individuals near the boundary, and so reduces mean density up to a few multiples of $\sigma_{D}$ away. Another choice is “reprising”, for which the random draw $\left(X,Y\right)$ is chosen conditionally so that $\left(x+\mathrm{\sigma X},y+\mathrm{\sigma Y}\right)$ stays within the range. (For more details of the consequences of boundary conditions, see Mazzucco et al. 2018).

Movement in practice often depends on the environment, of course: organisms tend to move within particular habitats, and barriers are ubiquitous on all scales. Small‐scale heterogeneity may be averaged out across the time scale simulated, and so incorporated (implicitly) in the movement kernel. However, large‐scale heterogeneity can be important. Movement on a heterogeneous landscape still relies on some way of randomly choosing nearby points, and hence a movement kernel. One way to incorporate this is discussed in Box 5.

### 4.1 Clumping

**FIGURE 3:**
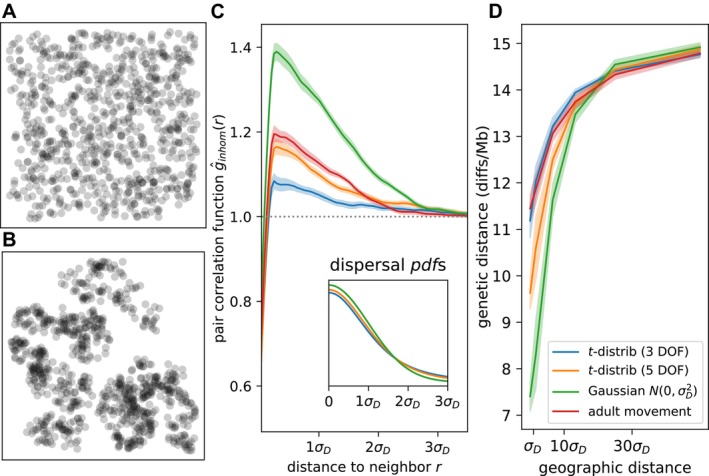
(A, B) Examples of simulations showing clumping (B) and not (A). (C) Dispersal kernel and movement type affects magnitude and spatial scale of clumping. Pair correlation functions (main panel) show density of pairs of individuals found a particular distance apart, relative to distances expected under a Poisson process (1.0; gray dotted line). Curves show the average across 50 independent time steps. Probability density functions (inset) for dispersal scales under the three dispersal kernels used: t‐distribution with 3° of freedom, t‐distribution with 5° of freedom, and Gaussian. Scale of t‐distributions is equal to the standard deviation of the normal distribution ($\sigma_{D}=0.3$). “Adult movement” scenario uses the Gaussian for dispersal as well as for movement at each time step. (D) Fat‐tailed dispersal and adult movement flatten genetic isolation by distance. Plots show mean genetic distance between pairs of individuals at increasing geographic distance, averaged across ten independent replicates. Figure S3 visualizes clumping quantified in (C, D); Figure S4 quantifies clumping visualized in (A, B).

In many species, reproduction is local, and so it tends to produce clumps that are spread out by movement and dispersal. Such patterns can be intriguing or puzzling and have real consequences for demography and genetic variation. Spatial clumpiness is sometimes visually obvious (as in Figure 3B), but more generally, the clumping tendency of individuals can be measured by spatial correlations.

One informative measure of clumping is the *pair correlation function*, which shows for each distance x how likely an individual is to have a neighbor at that distance relative to the average density. (See Baddeley et al. (2015) other useful descriptors of point data.) Concretely, the pair correlation function estimates the mean density at distance x away from an individual divided by the overall mean density, averaged across individuals; if the points are independently placed, it is constant at 1.0. Figure 3C shows pair correlation functions for simulations in which adults do not move and dispersal follows either a t or a Gaussian distribution, or in which both dispersal and adult movement are Gaussian (with $\sigma_{D}=\sigma_{V}$).

Individuals are more likely to be around a distance of $\sigma_{D}$ from each other than otherwise, but this tendency is reduced with more long‐distance dispersal (t dispersal with lower degrees of freedom; Figure 3C). In other words, a little long‐range dispersal reduces clumping. Note, however, that in these examples the scale of clumping is quite narrow: correlations only extend out to 2 or 3 multiples of $\sigma_{D}$. For another visualization of this relatively subtle clumping, see Figure S3. Unsurprisingly, adding adult movement ($\sigma_{V}\neq 0$) to a dispersal‐only model reduces correlations as well.

In spatial models, neighbors tend to be more related to each other than to distant individuals, a pattern known as “isolation by distance” (Wright 1943). The scale at which this correlation appears is determined by how far individuals move, and is also affected by the shape of the dispersal kernel: Figure 3C,D show that simulations with more long‐range movement—but with comparable mean dispersal scale—tend to have a weaker relationship between geographic and genetic distance. (See also Smith and Weissman 2023).

## 5 Mating and Other Pairwise Interactions

Mating is a crucial interaction for biological simulations, and there are numerous aspects and choices to consider. We probably don't want to simulate the detailed movement of individual pollen grains or the meanderings of a male moth seeking a female, and instead would like to skip to the realized outcome, that is, “choose a mate nearby.” (We follow the literature on mating systems in calling this “mating,” even when referring to plants or broadcast spawners).

As with dispersal (Section 4), it is easiest to specify what “nearby” means with a kernel: roughly, an individual can be chosen with probability proportional to the kernel. More concretely, if the kernel is ρ and the mating scale is $\sigma_{M}$, then an individual at x would assign weight $w_{i}=\rho \left(\left(x_{i}-x\right)/\sigma_{M}\right)$ to another individual at location $x_{i}$. The probability individual i is chosen is equal to $w_{i}$ divided by the sum of weights across all nearby individuals. This same method can be used for other individual‐to‐individual interactions, such as predators choosing prey individuals.

We can adjust the typical distance between mates with the mating scale, $\sigma_{M}$, which is directly analogous to parameters used to describe interaction ($\sigma_{X}$) and dispersal ($\sigma_{D}$) scales (Section 3), although each uses an underlying kernel in slightly different ways. Of course, the criteria determining potential mates for a given individual differ widely among species (Shuster 2003). Relevant questions about the mating system include: How often does selfing occur, and under what circumstances? Are sexes separate (dioecy/gonochory) or not (monoecy/hermaphrodity)? Are there distinct mating types or self‐incompatibility systems? An important note is that for dioecious species, calculations to determine stability of population density are easiest if done using only the reproducing sex.

BOX 4 **Interactions, and mating.**

The mechanism that SLiM uses to mediate most effects that some individuals have on others is called an “interaction type” (see Box 1). We used a symmetric interaction type in Box 1 to compute local density: every individual affected every other. Some interactions are not symmetric: we might, for instance, want each female to be able to find nearby males. To do this, we first set up a sex‐specific interaction (again using a Gaussian kernel, with standard deviation SM as in ${\text{sigma}}_{M}$):

21	initializeInteractionType(2, "xy", maxDistance=SM * 3, sexSegregation = "FM");22	i2.setInteractionFunction("n", 1.0, SM);

The sexSegregation parameter value of “FM” means that females will receive the interaction and males will exert the interaction; it is asymmetric. Then, we can use the interaction in a reproduction(NULL, “F”) block as follows (the NULL and “F” arguments imply that it applies to all females) to produce a single offspring:

23	mate = i2.drawByStrength(individual, 1);24	subpop.addCrossed(individual, mate, count=1);

This chooses a male mate from among the neighbors of the focal female, individual, with probability proportional to a Gaussian kernel with standard deviation SM. The interaction type itself guarantees that the chosen mate will be male. It is possible to set up other constraints on interaction types as well, such as a minimum or maximum ages, to represent other constraints on the reproductive eligibility of individuals.

### 5.1 Sex‐Specific Spatial Structure

**FIGURE 4:**
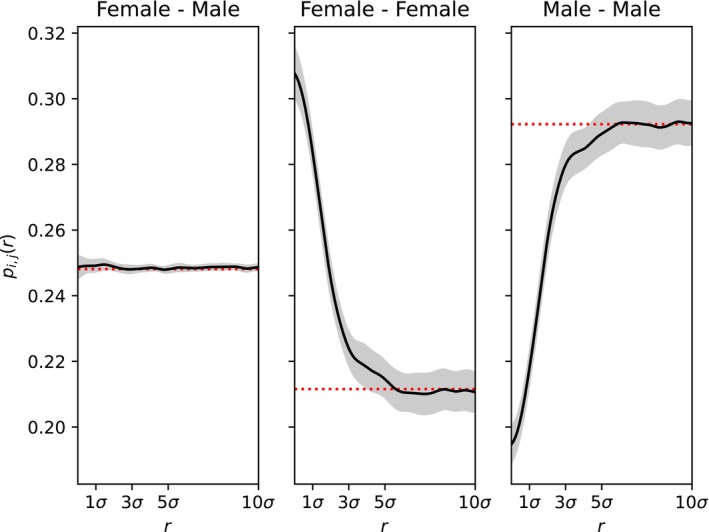
Dioecy generates female underdispersion, male overdispersion. Each panel shows the proportion of pairs of individuals at the distance shown on the horizontal axis that are either (left) a female and a male (center) both female, or (right) both male. Average and standard deviation of 50 independent simulation ticks are shown. Red dashed lines indicate expectation if individuals' locations were chosen uniformly. Note that the “Male–Female” proportion is be identical to the “Female–Male” proportion, so that twice the left panel plus the other two panels is equal to 1.

The combination of density dependence and mating system can have surprising consequences: for instance, in dioecious simulations, clustering is dependent on sex. To illustrate this, Figure 4 shows the *mark connection function* (Baddeley et al. 2015, §14.6.4.2) for female/male, female/female, and male/male neighbors, which shows the proportion of pairs of points at distance r that are one female and one male, two females, or two males, respectively. Curiously, we see that the probability that an individual's neighbor is of the other sex does not depend on that neighbor's distance. However, the probability of having a *same*‐sex neighbor *does* change with distance. As shown in Figure 4, within $3\sigma_{D}$ neighbors of a female are more likely than expected to be female, and nearby neighbors of a male are less likely to be male than expected.

Why does this happen? A simple reason is that we are modeling a dioecious scenario with no adult movement and where offspring are only generated by females and placed nearby. Local density‐dependent mortality means that all individuals tend to kill their neighbors, but only females can replace them. Correspondingly, the spatial range of a dioecious system where offspring disperse from a particular sex is determined by the range of that sex—others on the periphery cannot extend the range because offspring do not disperse from them. This is a disadvantage for colonizing new areas (Obbard et al. 2006) and may explain the spatial distribution of dioecious individuals away from a range front (Mirski et al. 2017).

Indeed, Shuster (2003) showed that female aggregation (as observed in Figure 4) is a universal consequence in mating systems with female choice (Shuster 2003, ch. 2). Other aspects of the simulation may differ by sex: for instance, sex‐biased dispersal is pervasive (Trochet et al. 2016) and generates detectable spatial patterns of genetic relatedness between sexes (Aguillon et al. 2017; Broquet and Petit 2009; Laporte and Charlesworth 2002). Social mating structures can induce sex‐biased dispersal and thereby create similar patterns (Pusey and Packer 1987; Hammond et al. 2005).

## 6 Maps: Spatial Heterogeneity

BOX 5 **Defining and manipulating maps.**

SLiM provides support for defining and manipulating spatial maps. We can read the values for a spatial map (say, of elevation) from a .csv file containing a rectangular grid of values using the code below:

25	mapValues = readCSV(“elevation.csv”).asMatrix();26	map = p1.defineSpatialMap(“elevation”, “xy”, mapValues);

(Images can also be read in as .png files.) If this is a low‐resolution raster then we may wish to smoothly interpolate it to higher resolution, done below with bicubic interpolation:

27	map.interpolate(factor=20, “cubic”);

Once we have this map, we can extract the values of the map at arbitrary locations—for instance, the elevations at which individuals live:

28 elevs = map.mapValue(inds.spatialPosition);

Further operations are available, including blurring and algebraic manipulations of the values. The dispersal method in Box 3 allowed individuals to move equally well in any direction. To guide movement with a map—for instance, to induce a preference for moving uphill, for this example where map values indicate elevation—we can use the map.sampleNearbyPoint() method:

29	inds = p1.individuals;30	pos = map.sampleNearbyPoint(inds.spatialPosition, INF, “n”, SM);31	inds.setSpatialPosition(pos);

This will move each individual to a new location sampled nearby and weighted by map value: if the original location is x, ρ is a Gaussian kernel (specified as type “n”) of width SD, as in $\sigma_{D}$, and the value of the map at location y is $m\left(y\right)$, then the new location is chosen with density proportional to $m\left(y\right)\rho \left(\left(y-x\right)/\sigma_{D}\right)$.

Most real populations are far from uniformly distributed in space, and in most cases, the underlying cause is thought to be environmental heterogeneity. Until this point, we have considered simulations of homogeneous landscapes only. Such “flat” landscapes are useful for developing models and/or theory, but incorporating aspects of real landscapes can make simulations more realistic. Spatial heterogeneity can be introduced simply by making some parameter of the model, such as fecundity or mortality (f or μ as defined in Section 2), vary across space—in which case we can visualize the parameter as a map. Such maps might represent specific environmental conditions, habitat boundaries, or abstract habitat quality.

Raster‐based images provide a convenient way to introduce a map of spatial heterogeneity into a simulation framework that cannot directly read geospatial data formats. A monochrome .png file consists of a rectangular grid of pixels with integer values between 0 and 255. These values can be shifted and scaled to lie within a useful range. See Case Studies (Section 9) for several examples of the use of images, and Box 5 for an example using .csv data. High‐resolution maps to use as source images are publicly available from various sources, including NASA's Earthdata platform (2024), ESA's Earth System Data Lab (2024), or PRISM Climate Group (2024). Open‐source tools for processing remote‐sensing data are also available (see Montero et al. 2023).

To use a raster‐based image, we must decide how the bounds of the image map onto geographic space. In general, we match the (rectangular) image with the (rectangular) spatial area to be simulated. The image is represented as discrete pixels, but we need to obtain values of the map at arbitrary locations (not just at the center each raster pixel). This can be done by either associating each pixel's value with the rectangle it would visually cover (as seen in Box 2), or associating each pixel's value with the corresponding cell's midpoint and interpolating between.

**FIGURE 5:**
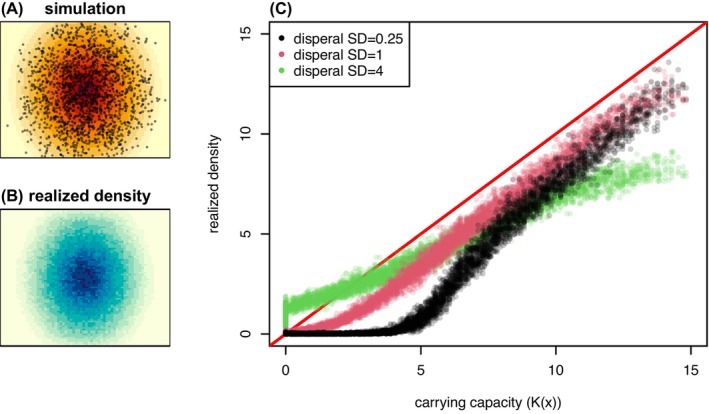
A spatial simulation using a heterogeneous map of carrying capacity generated from an image file (A) A representative time step during the simulation, with each of the roughly 1500 individuals shown as points and the map of carrying capacity, $K\left(x\right)$, shown in the background. Red hue indicates greater $K\left(x\right)$ in the pixel (B) A map of realized density, averaged over $10^{4}$ time steps. Blue hue indicates greater individual density in the pixel (C) Comparison of realized density to carrying capacity from three different runs using different dispersal scales: Each point shows the realized density in one of the pixels of the map shown in (B) plotted against the value of $K\left(x\right)$ in the center of the pixel, for three values of $\sigma_{D}$ (labeled “SD” in the legend). The dimensions of the map are 25 × 20, and the interaction scale is $\sigma_{X}=0.3$. The simulation has Ricker regulation of fecundity: The expected number of offspring of an individual at location x is $\exp\left(-\frac{n\left(x\right)}{K\left(x\right)}-1\right)\mu /\left(1-\mu \right)$, where μ is the (fixed) probability of survival, $n\left(x\right)$ is the local density at x, and $K\left(x\right)$ is obtained from the value of the image at x.

In the simple example shown in Figure 5, an image represents the altitude map of a mountain, with darker red shades indicating higher elevation. The local carrying capacity, $K\left(x\right)$, is modified by the value of the map at x so that high‐elevation locations can support more organisms. Comparing the provided altitude map (background of Figure 5A) with a summary of realized average density (Figure 5B), demonstrates that population density roughly matches the underlying elevation map.

A more precise comparison (Figure 5C) shows that $n\left(x\right)$, the local density at x defined in Equation (1), is not exactly $K\left(x\right)$. In most situations, the equilibrium density is below $K\left(x\right)$. This is probably for two reasons: first, stochasticity usually reduces equilibrium density (see Section 7.3 and Appendix B.4); second, since the mountain is conical, most locations x are surrounded by more low elevation area (where K is lower than $K\left(x\right)$) than higher area (where K is higher than $K\left(x\right)$), making the net flux of migrants at x negative.

The degree of deviation of $K\left(x\right)$ from $n\left(x\right)$ can depend on other factors such as $\sigma_{D}$. When dispersal is short ($\sigma_{D}=0.25$, black points), areas with carrying capacity below about 5 individuals per unit area are not self‐sustaining. (See Appendix B.3 for more discussion.) On the other hand, with long‐range dispersal ($\sigma_{D}=4$, green points), the overall relationship between density and carrying capacity is flattened as offspring from high‐fecundity areas end up across the entire range. However, most offspring are still produced near the top of the mountain, and lower elevations are maintained by source‐sink dynamics.

BOX 6 **Adaptive empirical tuning for emergent parameters.**

We often want a simulation to match a given estimated or observed density. However, this is not as simple as setting the value of K (local carrying capacity, introduced in Section 2) in the code, because population density is an *emergent* quantity—a complex consequence of births, deaths, movement, and local interactions. Fortunately, matching even emergent quantities is possible in SLiM. The code in this box dynamically adjusts parameters to make population density match a desired value. The same general technique may be applied to other quantities such as mean age or degree of clustering.

We first define a global parameter ADJ that will be adjusted:

32	defineGlobal(“ADJ”, 1.0);

Then we modify the density regulation code from Box 1 so that ADJ adjusts the carrying capacity:

33	inds = p1.individuals;34	density = 11.localPopulationDensity(inds);35	u = density / ((1 + f) * K);36	inds.fitnessScaling = 1 / (1 + ADJ * f * u);

In each time step, ADJ is updated by a factor $\exp\left[\alpha \left(Y-K\right)\right]$, where Y is global population density (population size divided by total area) and α is an update rate (e.g., α=0.01):

37	obsDensity = p1.individualCount / (WIDTH * HEIGHT);38	defineGlobal(“ADJ”, ADJ * exp((obsDensity - K) * ALPHA));

When Y>K, we want the density to be lowered in the next cycle, so ADJ is increased, decreasing carrying capacity. On the other hand, when Y<K, ADJ is decreased so that we get a higher realized global population density Y in the next cycle.

Importantly, this tuning should stop after an initial “burn‐in” period, and the appropriate value for ADJ, once found, should be hard‐coded into the final model. If changes occur in the simulation that affect population size (e.g., a reduction in habitat), this adaptive code will force the density back to the chosen value, which is generally not desirable—the population size should, in fact, change in response to such changes in the simulated conditions.

## 7 Density Dependence, Life History, and Stochasticity

In our initial model, we used Beverton–Holt density‐dependent feedback on mortality to control global population size (Section 2.2). Here we give examples that have the same Beverton–Holt form for the net effect of density on population regulation but differ in other ways.

### 7.1 Fecundity Regulation

The Beverton–Holt density‐dependent regulation in Equation (4) has the probability of death increase with local density while fecundity stays constant. Alternatively, we can set the probability of death to a constant: $\mu \left(u\right)=\mu_{0}$, and then solve for $f\left(u\right)$ to obtain the Beverton–Holt form $F\left(u\right)=\alpha \left(\left(1+a\right)/\left(1+\mathrm{au}\right)-1\right)$. (It turns out we will need the scaling factor 0<α<1 to make a model with positive birth rates and death probabilities between 0 and 1.) Then, fecundity should depend on scaled density u as follows:

(5) $$f\left(u\right)=\frac{1}{\left(1-\mu_{0}\right)}\left(\alpha \frac{\left(1+a\right)}{\left(1+\mathrm{au}\right)}+\left(\mu_{0}-\alpha \right)\right).$$

This functional form is shown in the middle panel of Figure 1.

### 7.2 Compensatory Regulation on Juvenile and Adult Mortality

To stabilize the population, the effect of density on *net* reproductive rate must be negative, but the effects on individual demographic components (such as birth or death) can be positive, as long as they are compensated for by other components. For instance, the probability of survival $1-\mu \left(u\right)$ can *increase* with density as long as fecundity $f\left(u\right)$ decreases faster. One way to do this is to set

(6) $$f\left(u\right)=\frac{f_{0}}{1+\mathrm{bu}}\;\text{and}\;1-\mu \left(u\right)=\frac{1+F\left(u\right)}{1+f\left(u\right)}.$$

where $F\left(u\right)=\alpha \left(\left(1+a\right)/\left(1+\mathrm{au}\right)-1\right);$ that is, the Beverton–Holt form again. This produces valid survival probabilities if $f_{0}\geq \mathrm{a\alpha}$. Depending on the choice of the parameters, $f\left(u\right)$ may increase or decrease with u, and is not necessarily monotone. One choice of valid parameters is shown in the rightmost panel of Figure 1.

Our three examples (4), (5), and (6) (illustrated in Figure 1) might all be reasonably called “Beverton–Holt” models, although they differ substantially in the underlying mechanism of regulation. Although they have similar behavior around equilibrium density, they have quite different life‐history implications. Most strikingly, in the first model, mortality increases with density; in the second, mortality is constant, while in the third, mortality decreases with density. There are corresponding differences in age structure among the models (as shown in Figure S5), although the dynamics of total population size are similar. More examples along these lines are given in Appendix C.

### 7.3 Stochastic Effects

Despite all this theory, in practice, equilibrium density is usually not K—it is often lower. (See Figure 5 and Figure S6, for instance.) This is due to various forms of stochasticity. One is random lack of mates (when $N_{M}$ is small); another is local extinction caused by temporal population fluctuations (when $N_{X}$ is small). These effects can be quite troublesome when setting up computational experiments across a range of parameters, especially if we want constant total population size.

Apart from those issues, the most common reasons for a significant discrepancy between the realized density and the “desired” density (set by K) have to do with when and how density is measured. First: *when* is density measured? In each time step, density increases after birth and decreases after death; K can match at most one of these times. Second: *where* is density measured? We naturally look at total population size divided by total area; however, on average individuals experience a higher density, since they themselves count toward their own local density. So, the correct comparison is of K to local population density averaged across the location of all individuals. Since this oversamples areas of higher density, this density will be higher than “total population size divided by area.”

All of these issues are discussed in much greater detail in Appendix B. For practical reasons, it often suffices to simply be aware that population density is fundamentally an emergent property, determined in complex ways by nearly all parts of the life cycle. If a precise total population size is desired in a particular simulation, a simple solution is to adjust some parameter (e.g., the birth rate) until the desired value is achieved (see Box 6), but of course, that will alter the dynamics of the simulation in other respects.

**FIGURE 6:**
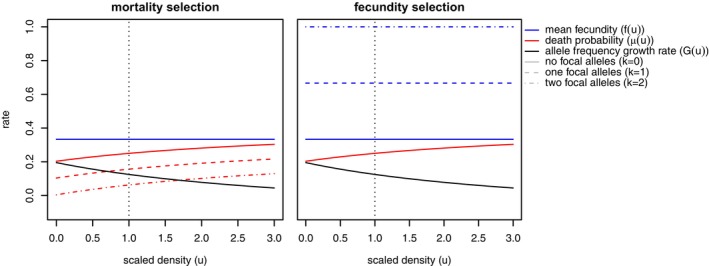
Density dependence of for two methods of selection: On (mortality) or (fecundity), as described in Equations (8) and (10) with s=0.25. Line types show the mortality (red) or fecundity (blue) for individuals carrying k=0, 1, or 2 copies of the beneficial allele. The mean growth rate of the beneficial allele when rare (black line; $G\left(u\right)$ from Equation (9)) is the same for both types of selection, at all densities. The vertical dotted line is at scaled density u=1. Other parameters are as in Figure 1.

## 8 Natural Selection

It turns out that there are many different ways to implement natural selection in an individual‐based simulation that all map to the same abstract models from population genetics, yet produce distinct outcomes. Modelers may choose to have an individual's genotype affect its choice of mate, its preference for certain habitats, its longevity, or any number of other life‐history traits. These effects may differ across a landscape, leading to local adaptation. In this section, we demonstrate selection that acts on birth and death, building on Section 2. Just as we could regulate population size in our individual‐based simulation through birth rate, death rate, or both, one may achieve the same increase in “fitness” by increasing fecundity or decreasing mortality, and we will see that the selected allele thus affects equilibrium population density. The resulting models might be classified as having “hard” selection (Wallace 1975); “soft” selection might act through increased or decreased chance of being chosen for mating (and would usually cause little to no change in equilibrium population density). For a view of the literature on these ecological‐evolutionary models, see Mallet (2012) or Travis et al. (2023).

Much of population genetics selection theory is expressed in terms of the “selection coefficient” of a variant, usually denoted s, which is usually defined in the context of a Wright–Fisher model as the change in relative probability it confers on the individual to be “selected” to provide offspring to the next generation. The point in the life cycle when the variant confers its selective effect is known to produce differences even in nonspatial models (Bodmer 1965; Nagylaki and Crow 1974), and so analogies between the dynamics of such alleles and those in a Wright–Fisher model are necessarily approximate. This is important when attempting to match simulation results to theory: just because a variable that affects survival or fecundity is named s does not mean that using its value in expressions derived from the Wright–Fisher model correctly predicts the probability of establishment, mean frequency, or other classical quantities, even in a nonspatial simulation.

Below, we demonstrate two models in which selection acts on either mortality or fecundity. We choose our parameter s so that a variant's frequency changes on average by a factor of 1+hs per time step when rare, where h is the dominance coefficient. (Note that this may not be the best definition for use with classical formulas, which often measure change per generation.) For a historical and philosophical review of definitions and measures of selection see Endler (1986). Implementation in SLiM is described in Box 7.

### 8.1 Defining Allelic Growth Rate

Recall from Section 2 that $f\left(u\right)$ and $\mu \left(u\right)$ are, respectively, the per‐capita mean number of offspring and probability of death per time step for an individual experiencing scaled density u. For selection, these vital rates should depend on the individual's genotype. So, let $f_{k}\left(u\right)$ be the mean fecundity of an individual with k copies of a focal allele, and similarly $\mu_{k}\left(u\right)$ the probability of death. When the focal allele is rare, most of the population use $f_{0}\left(u\right)$ and $\mu_{0}\left(u\right)$, but a few individuals use $f_{1}\left(u\right)$ and $\mu_{1}\left(u\right)$. So, then the per‐capita rate at which the number of focal alleles grows when at scaled density u in an outcrossing species is

(7) $$G\left(u\right)=\left(1-\mu_{1}\left(u\right)\right)\left(1+\left(f_{0}\left(u\right)+f_{1}\left(u\right)\right)/2\right)-1.$$

This is almost the same as the expression for $F\left(u\right)$, the expected change in the number of *individuals* from Equation (2), except that the fecundity term is $\left(f_{0}\left(u\right)+f_{1}\left(u\right)\right)/2$; this is because the offspring of a heterozygous parent will only inherit the focal allele from that parent half the time.

### 8.2 Parameterizing Selection

First we consider mortality‐based selection. Suppose an additive allele (with h=1/2) increases survival by s/2 per copy (k), so that

(8) $$1-\mu_{k}\left(u\right)=\left(1-\mu \left(u\right)\right)\left(1+\mathrm{ks}/2\right).$$

If the allele doesn't affect fecundity (meaning $f_{k}\left(u\right)=f\left(u\right)$), then we plug Equation (8) into Equation (7) with k=1 to find that per‐capita allelic growth rate in an outcrossing species is

(9) $$G\left(u\right)=\left(1-\mu \left(u\right)\right)\left(1+f\left(u\right)\right)\left(1+s/2\right)-1.$$

We have set things up here so that s means what we want: at the population's equilibrium density, the allele's growth rate when rare is $G\left(1\right)=s/2$.

Now suppose that the allele increases fecundity and does not affect survival (meaning $\mu_{k}\left(u\right)=\mu \left(u\right)$). We define

(10) $$f_{k}\left(u\right)=\left(1+f\left(u\right)\right)\left(1+\mathrm{ks}\right)-1.$$

With this form for fecundity selection, the allelic growth rate when rare is the same as the rate we calculated for mortality‐based selection (Figure 6). We can check this by plugging in our fecundity expression (10) into Equation (7) with k=1 and verifying that we get the same expression as Equation (9). Therefore, at equilibrium, the per‐capita growth rate of the number of alleles is again s/2. Roughly speaking, each allele increases fecundity by s, rather than the s/2 in Equation (8) for mortality selection, because here the effects of fecundity are only affecting half of the parents (the offspring‐bearing ones).

### 8.3 Spatial Selection Mechanisms in Practice

**FIGURE 7:**
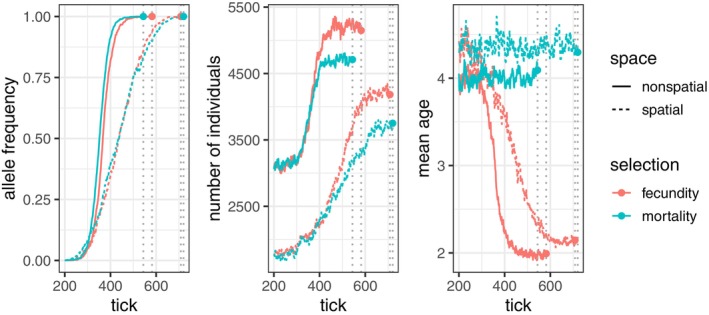
Allele frequency (left), population size (center), and average individual age (right) over time in spatial and nonspatial nonWF simulations. Curves end at the time the selected allele is fixed (dotted vertical lines).

Above we laid out deterministic, large‐population‐size theory that suggests that a variant that affects survivorship or fecundity might have similar frequency dynamics. However, in practice, there are some meaningful differences. Figure 7 shows selective sweeps in spatial and nonspatial simulations. All simulations use density‐dependent feedback on mortality (via Equation (4)), but selection acts on either fecundity (via Equation (10)) or mortality (via Equation (8)). In nonspatial simulations, the global density (total number of individuals divided by total area) is substituted for local density when computing the probability of survival.

Some features of the sweep experiments are expected. Selective sweeps progress more slowly in spatially structured populations. The allele‐frequency trajectories of *de novo* mutations under either mortality‐ or fecundity‐based selection (Figure 7A), at first increase at the same speed in spatial and nonspatial sweeps, as predicted (i.e., $G\left(u\right)$ is the same for both when the allele is rare). However, the selection mechanism introduces a few differences. As the beneficial allele fixes, equilibrium population density increases by a greater amount under fecundity‐based selection (Figure 7B) than mortality‐based selection. To understand why this is, consider the population after the fixation of the beneficial allele: all individual birth and death rates are now $f_{2}\left(u\right)$ and $\mu_{2}\left(u\right)$. We are at a new equilibrium density $u_{*}$, which occurs when net per‐capita change in population size is zero. Solving Equation (2) with our post‐fixation vital rates $\left(F\left(u_{*}\right)=0\right)$, the new equilibrium density $u_{*}$ is $u_{*}=1+s\left(1+\frac{1}{f_{0}}\right)$ for mortality‐based selection, and $u_{*}=1+2s\left(1+\frac{1}{f_{0}}\right)$ for fecundity‐based selection. In other words, the increase in equilibrium density for fecundity‐based selection should be roughly twice that of the increase in mortality‐based selection.

Fecundity‐based selection also causes the mean individual age to drop as the beneficial allele increases in frequency (Figure 7C). Population size increases as the fecundity‐based sweep progresses, but because the beneficial allele does not confer protection against mortality, individuals are subject to the negative effects of increased population density.

Further differences between the spatial and nonspatial simulations are likely due to the spatial density regulation processes that cause realized population sizes in spatial simulations to be smaller than in their nonspatial counterparts. (See Section 7.3 and Appendix B.4 for discussion).

BOX 7 **Selection.**

In nonWF SLiM models, the “fitness” property of an individual is the probability that the individual survives to the next time step. To make each mutation affect survival by a factor of S_MORT, we simply declare:

39	initializeMutationType(“m1”, 0.5, “f”, S_MORT);

(The factor of 0.5 is the dominance coefficient: heterozygotes will have fitness multiplied by 1 + S_MORT*0.5, and homozygotes by 1 + S_MORT).

To have the same type of mutations also affect fecundity with selection coefficient S_FEC, we need to account for genotype when setting up offspring numbers (as in Box 1):

40	indiv_s = 2*S_FEC * inds.countOfMutationsOfType(m1) / 2;41	inds.tag = rpois(length(inds), mean=FECUN * (1 + indiv_s) + indiv_s);

As written, the effects of any mutation of type m1 on fecundity are additive across loci and copies; other arrangements are possible.

## 9 Case Studies

How can we combine all the strategies we have seen thus far into a useful model of a living system? Here we illustrate how the spatial modeling framework we have introduced can be used to model complex scenarios such as (1) temporal change, (2) complex life cycles, (3) continental‐scale systems, and (4) competition for resources.

We motivate each scenario with an organism. The scenarios are not meant to be complete or accurate models of the population biology of these organisms; rather, they illustrate how to apply the concepts presented in this paper to a study system. Additional methodological information for each vignette can be found in Appendix E and our code example repository https://github.com/kr‐colab/spatial_sims_standard.

### 9.1 Changes Over Time: Pikas and Environmental Change

Modeling spatial heterogeneity (as introduced in Section 6) makes simulations more realistic and informative, but sometimes *temporal* change in landscape variables is just as important. Here we explore how both seasonal fluctuations and globally rising temperatures affect the population dynamics of an alpine organism: the pika.

Pikas (*Ochotona daurica*) are adapted to mountainous habitat at relatively high elevation and cannot survive the extreme heat (or cold) at lower (or higher) elevations (Beever et al. 2010). Such ecology makes pikas potentially vulnerable as global temperatures are expected to increase over time.

#### 9.1.1 Modeling Approach

We incorporate three types of temporal change in temperature: (1) within‐year seasonal change, (2) random, autocorrelated fluctuations between years, and (3) steady global temperature rise. Because temperature varies with elevation and elevation varies dramatically in mountainous regions, we model temperature as a function of elevation. We used a topographic map for a region of Rocky Mountain National Park in Colorado to calculate temperatures for each point in space and time during our simulation.

We connect temperature to fitness by killing offspring that are exposed to temperatures beyond the minimum ($-5^{\circ }C$) or maximum ($28^{\circ }C$) sustained by pikas (Beever et al. 2010). Each tick of our simulation represents 1 year. To account for *within‐*year seasonal variation (i.e., winter cold and summer heat), we narrow the viability range by the yearly variation in temperature ($s^{\text{seas}}$), defining the probability of survival of a pika at location x in year t to be

$$1-\mu \left(x,t\right)=\left\{\begin{array}{ll} 0, & \text{if}\;T\left(x,t\right)<\left(-5+s^{\text{seas}}/2\right)\\ 0, & \text{if}\;T\left(x,t\right)>\left(28-s^{\text{seas}}/2\right)\\ \frac{1}{1+\mathrm{fu}\left(x\right)}, & \text{otherwise} \end{array}\right.$$

where the last term is our usual Beverton–Holt mortality regulation with local scaled density $u\left(x\right)=n\left(x\right)/K$ (see Equation (4)). In this equation, $T\left(x,t\right)$ represents the midpoint of seasonal temperatures in year t, defined below, and the within‐year variability $s^{\text{seas}}$ broadens the temperatures experienced by the pikas around that midpoint. For example, if $T\left(x,t\right)$ were $-4^{\circ }C$ (inside the viability range), but seasonal temperatures varied by $4^{\circ }C$, the winter would be $-6^{\circ }C$—cold enough to kill.

Between‐year temperature changes are modeled by the temperature function

$$T\left(x,t\right)=T^{\text{elev}}\left(x\right)+T^{\text{fluct}}\left(t\right)+0.016t$$

which combines elevation‐related temperature change $T^{\text{elev}}$, correlated year‐to‐year fluctuations $T^{\text{fluct}}$, and global directional climate change.

$T^{\text{elev}}\left(x\right)$ is calculated from the elevation map following fitted models from Antonio‐Juan Collados et al. (2020). To create autocorrelated year‐to‐year fluctuation, we let $T^{\text{fluct}}\left(0\right)=0$ and for t≥1,

$$T^{\text{fluct}}\left(t\right)=p^{\text{fluct}}T\left(t-1\right)+\epsilon \left(t\right)$$

where $\epsilon \left(t\right)$ is Normally distributed noise with mean zero and standard deviation ($s^{\text{fluct}}$). The parameters $p^{\text{fluct}}$ and $s^{\text{fluct}}$ are known as persistence and shock, respectively, and determine how correlated the noise is between years. Finally, we increase the global temperature over time by adding 0.016 C per year (Foster and Rahmstorf 2011).

#### 9.1.2 Observations and Extensions

**FIGURE 8:**
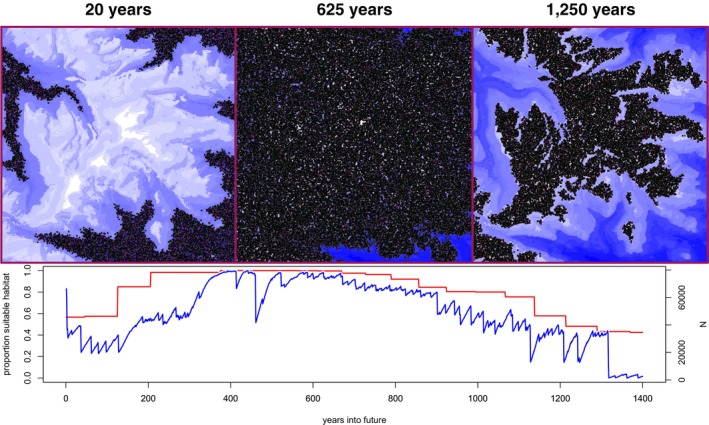
Pika simulation. (Top) Screenshots of individual spatial positions (black) at different time points. The background image shows elevation, where blue and white correspond to lower and higher elevation, respectively. (Bottom) The blue line shows population size over time; the red line shows the proportion of habitable space before the addition of random noise.

The resulting simulation is a habitat suitability model in which the population's geographic distribution moves toward higher elevation as the global temperature increases (Figure 8).

We found that if the magnitude of random variation around the expected annual temperature is large ($s^{\text{fluct}}=5^{\circ }$C) the probability of extinction increases significantly, particularly for early generations. In other words, one or a few bad years was devastating for pika populations. This result may provide a useful lesson: even if a species appears to be thriving, the long‐term success of the species is not guaranteed.

Surprisingly, simulated population size increased in the intermediate‐term future, since for the first few hundred years, habitat losses at lower elevation were more than compensated for by habitat gains at higher elevation. This illustrates that results from simulation on a specific map may not be generalizable.

Here, we use an empirically informed linear trend with Gaussian noise as our global temperature change schedule. An alternative strategy for simulating temperature changes would be to pre‐process multiple temperature maps reflecting different years and continually feed the simulation new maps over time. Such maps could be generated directly from a climate model rather than using a direct function of elevation.

### 9.2 Life Cycle Stages: Mosquitos in Burkina Faso

So far, our models have not taken life cycle stages into account; individuals have been able to mate immediately after they are born, and their survival has not been age‐dependent. However, for many organisms, their patterns of mobility, competition for resources, and mating capability are age‐dependent. Here, we demonstrate how to simulate a population with distinct juvenile and adult phases. Specifically, we set up a simulation of mosquitos in Burkina Faso in West Africa, inspired by North et al. (2019).

#### 9.2.1 Modeling Approach

In this model, individuals begin life as juveniles, and mature into adults after a fixed time span. Population regulation and individual behavior depends on the life stage. For adults this is a constant survival probability of 0.875 per day (time step), independent of the landscape map. The population is regulated by density‐dependent survival of the *larvae*, which varies across the map.

Following the model in North et al. (2019), we set the carrying capacity of larvae based on water availability. Larval carrying capacity is only non‐zero at the outlines of water features extracted from GIS data of inland water in Burkina Faso. There, the carrying capacity fluctuates seasonally to mimic rainy and dry seasons:

$$K\left(x,t\right)=K_{\text{base}}\left(x\right)+K_{\text{rain}}\left(t\right)$$

where $K_{\text{base}}\left(x\right)$ is obtained from the map of inland water, and $K_{\text{rain}}\left(t\right)$ is a sinusoidal function with a period of 365 days and a minimum value of 0.

We then use a Beverton–Holt form for the survival probability of larvae. Since there are ten age classes for larvae (and survival of each depends on the total number across all ages), parameterizing the model so that local density is (roughly) K involves solving a system of equations (in a matrix model) described in Appendix E.2.

A female adult mosquito mates with an adult male within the maximum mating distance and lays eggs by sampling a location within her dispersal radius weighted by carrying capacity. Each day, adult mosquitos move by a random displacement sampled from a Gaussian distribution, whereas larvae do not move from their original location until they mature.

#### 9.2.2 Observations and Extensions

**FIGURE 9:**
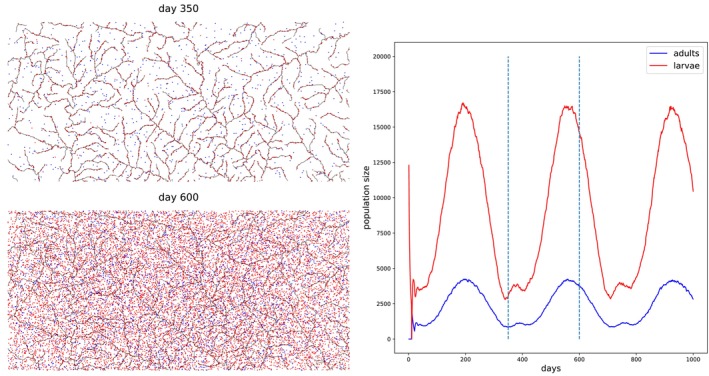
(Left) Adult (blue) and larval (red) populations on the river map of Burkina Faso, at two time points during the year: In the dry season (day 350), larvae can only survive in bodies of water, while in the wet season (day 600), larvae can survive in many places. (Right) Adult and larval population sizes oscillate with the seasonal cycle. Vertical lines indicate the time points plotted on the left.

This model simulates a mosquito population with a structured life history. The population size of larvae and adults fluctuates periodically, following precipitation levels with a slight lag (Figure 9). Recall that adult mortality is simply constant, so the periodic fluctuations of adults are mediated through larval carrying capacity dynamics.

There are a few immediate extensions to our simulation. Here, we did not distinguish between perennial and fluctuating water sources. Simulating water sources that appear and disappear (adding a t argument to $K_{\text{base}}\left(x\right)$) could result in extinction dynamics such as those observed in the pika example above. Similarly, the amount and duration of rainfall could be location‐dependent (adding an x argument to $K_{\text{rain}}\left(t\right)$).

We set the maximum of the $K_{\text{base}}$ and $K_{\text{rain}}$ functions to 0.002 individuals per square meter, which is probably much lower than in nature, to keep the memory usage of our simulation low. To run a simulation with a realistic (huge) number of mosquitoes we would need to make some efficiency improvements. Fortunately, an example of the necessary techniques follows.

### 9.3 Continental‐Scale Systems: Invasion of the Cane Toads

There is a perception that individual‐based spatial models are so slow that they are meaningfully limited in the population size that can be modeled. Our goal with this vignette is to demonstrate that this is not necessarily the case by showing how to model large‐scale populations and landscapes with relative ease and efficiency.

Cane toads ( *Bufo marinus* or *Rhinella marina* ) are native to Central and South America and were intentionally introduced to the Northeast coast of Australia in 1935 as a pest‐control measure. Since their introduction, cane toads have experienced explosive population growth, with hundreds of millions of individuals spreading over several million square kilometers, resulting in considerable negative economic and ecological impacts (Shine 2010; Urban et al. 2008).

#### 9.3.1 Modeling Approach

We use information about the biology of cane toads, when available, to parameterize the model. Toad population densities in established populations have been estimated around 8000 per square kilometer (Freeland 1986). Based on this, and then tailored heuristically over several trial runs to be computationally feasible and to produce a reasonably realistic pattern of range expansion, we settled upon a local carrying capacity of 1000 per square kilometer. Telemetry data shows cane toads can travel up to 0.2 km per day (Shine et al. 2021). In order to interpret simulation time steps roughly as years, we set the spatial scale parameters $\sigma_{D},\sigma_{X}$, and $\sigma_{M}$ to 20 km. For dispersal, we use a Student's t‐distribution, which provides more long‐range dispersal events than a Gaussian kernel (Figure 3C).

We model survival probability as a function of precipitation due to its similar distribution to empirical occurrence data, which seems reasonable given cane toads' known sensitivity to moisture conditions (Child et al. 2009; Cohen and Alford 1996). Specifically, we multiplied our typical density‐dependent survival probability $1-\mu \left(u\right)$ by $1/\left(1+\exp\left(-\left(\alpha +\mathrm{\beta P}\left(x\right)\right)\right)\right)$, where α and β control the intercept and slope of the survival curve, and $P\left(x\right)$ is the amount of yearly precipitation at location x.

The invasion began with 10,000 individuals (though the number of individuals actually released in 1935 was likely greater (Shine et al. 2020)) at locations randomly sampled from the first 4 years of the observed occurrence data.

Indeed, mating and interaction neighborhoods were large, which initially led to prohibitively long runtimes. To make the simulation more efficient, we followed the approach described in Box 8, modified to measure the local density of individuals per unit of *habitable* area. Mate choice was also modified as described in Box 9.

#### 9.3.2 Observations and Extensions

**FIGURE 10:**
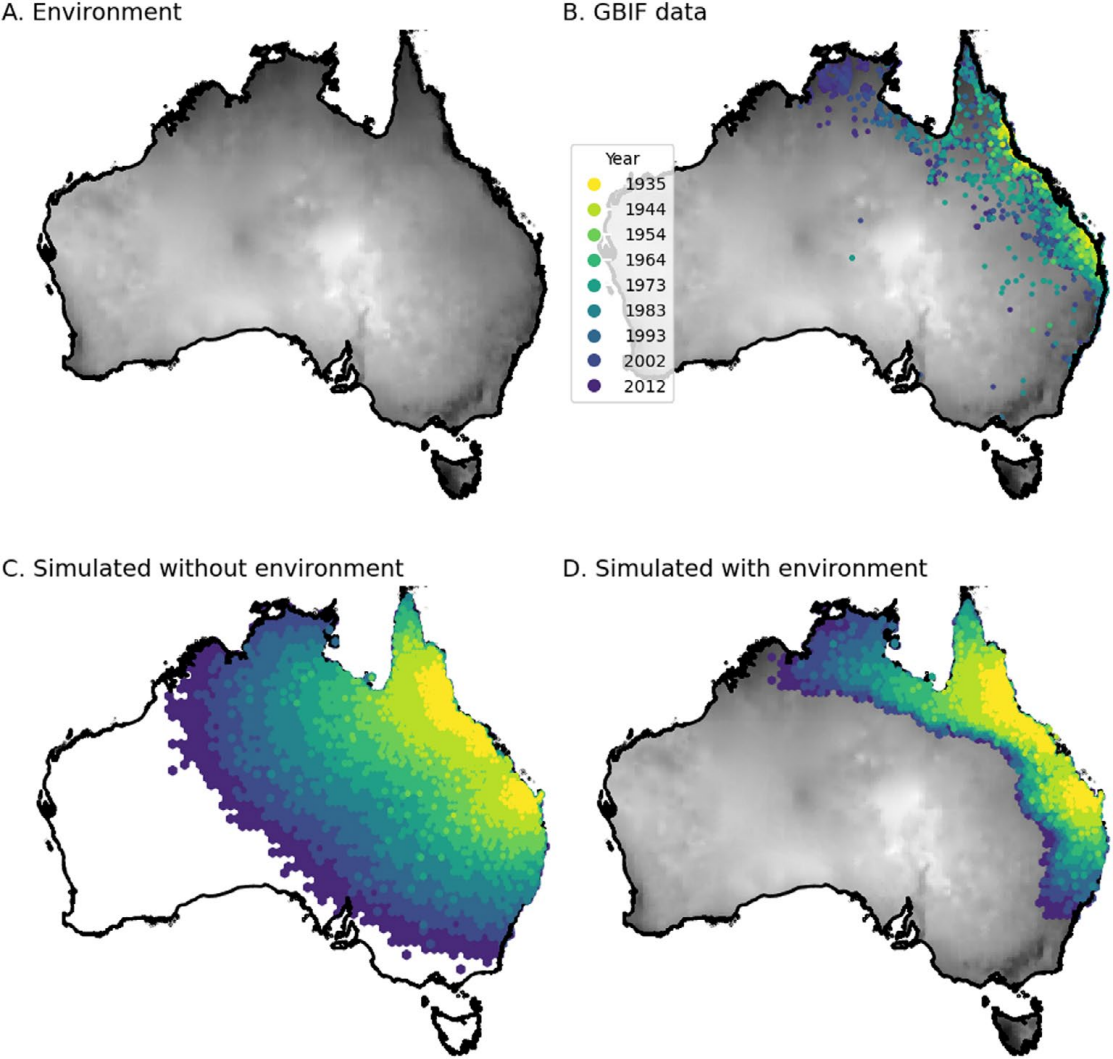
Simulating the cane‐toad invasion with and without and effect of annual precipitation on survival: (A) Map of Australia shaded by annual precipitation (kg m^−2^ year^−1^); (B) observed distribution of cane toads from Global Biodiversity Information Facility (GBIF); (C) simulation of cane‐toad invasion without effect of annual precipitation on survival; (D) same as (C), with effect of annual precipitation on survival.

We were able to approach the true spatial and population scale of the cane‐toad invasion, with a final census size of about 120 million individuals, nearing the estimated modern census size of Australian cane toads (WWF Australia 2019), after running for about 5 days and using 200 GB of RAM at maximum. We visually compared empirical occurrence data for cane toads to our simulations, with and without annual precipitation's effect on survival (Figure 10). While there are obvious differences in densities and locations between the simulated and observed data, it is clear that modeling annual precipitation's effect on survival greatly improves the likeness.

The approach described here is similar to classical niche modeling (Peterson 2001), which has been used extensively to model cane‐toad distributions (Shine 2010), but is somewhat simplified by only using one environmental variable. This would be a straightforward extension: multiple environmental variables could be combined into a single habitability map for use in the model, with no impact on runtime performance. The benefit of this vignette's approach over previous approaches is the way that it combines information from the explicit individual‐based simulation and environmental data, producing a simulation at a realistic scale with respect to both landscape size and population size.

BOX 8 **Using maps for faster spatial interactions.**

In Box 1, we estimated the local population density for each individual with Equation (1), using the localPopulationDensity() method. For each individual, this method sums the “interaction strength” (i.e., kernel density) for every other individual within the provided maximum distance. So, if the total number of individuals is T and the typical number of neighboring individuals is $N_{X}$ (defined in Section 3), then the complexity of this operation is ${\mathrm{TN}}_{X}$. If each individual has a large number of neighbors, this can be quite costly. However, if the number of neighbors is large, it should work just as well to (a) create a (discretized) map of local density, and (b) look up the value of the local density experienced by each individual on that map. Map lookup is quick, so if the cost of creating the map is smaller than ${\mathrm{TN}}_{X}$, we will have a more efficient model.

We can create a map whose value at x is (approximately) given by Equation (1) in two steps: (1) use summarizeIndividuals() to measure the number of individuals per unit area in each cell in a grid (see Box 2), and (2) smooth() this map using the appropriate kernel, so that the density value at a given point in the resulting map depends upon an appropriate weighted average of the individuals per unit area across nearby cells of the grid. Using the same kernel as in Box 1:

42	grid_dims = ceil(2 * p1.spatialBounds / SX);43	raw = summarizeIndividuals(p1.individuals, grid_dims, p1.spatialBounds,44		operation=”individuals.size();”, perUnitArea=T);45	density_map = p1.defineSpatialMap(“density”, “xy”, raw);46	density_map.smooth(SX * 3, “n”, SX);47	defineGlobal(“DENSITY”, density_map);

Then, we can modify the code of Box 1 to use the map instead:

48	inds = p1.individuals;49	density = p1.spatialMapValue(DENSITY, inds.spatialPosition);50	u = density / ((1 + f) * K);51	inds.fitnessScaling = 1 / (1 + f * u);

This obtains (nearly) the same value as would localPopulationDensity() if the resolution of the map should be finer than the scale over which the density kernel changes. In this example, that scale is SX, as in $\sigma_{X}$, so we have ensured that the map has cells of size smaller than SX/2. The approximation is examined in Appendix F.

BOX 9 **Using maps for faster mate choice.**

A similar problem as in Box 8 arises when choosing mates: even though only one mate needs to be chosen, the underlying operation is of order ${\mathrm{TN}}_{M}$, where T is the global population size. The same map of density can be used to solve this problem as well: instead of choosing an individual with probability proportional to a kernel, it is (nearly) equivalent to: (1) choose a point in space nearby, with probability proportional to the map multiplied by the kernel, and then (2) take the individual closest to that point. Recall the number of possible mates scales as $N_{M}=4\sigma_{M}^{2}K$ from Section 3. This implies that the number of neighbors grows linearly with K. Thus, we can keep the number of potential mate roughly constant regardless of how large local density is by rescaling the maximum distance in the InteractionType used for mate choice by $1/\sqrt{K}$ from the code in Box 4:

52	initializeInteractionType(2, “xy”, maxDistance=5/sqrt(K), sexSegregation = “FM”);53	i2.setInteractionFunction(“f”, 1.0);

We have set the maximum distance in the interaction kernel to be a value that should give us around 25 neighbors for each individual; however, if density varies significantly across the landscape, this may make some individuals in low density areas fail to mate. Then, we choose the mate as follows:

54	mate_location = DENSITY.sampleNearbyPoint(individual.spatialPosition,55		3*SM, “n”, SM);56	mate = i2.nearestNeighborsOfPoint(mate_location, p1, 1);

Here the specification of a Gaussian mate choice kernel with standard deviation SM has moved from the definition of the InteractionType to the sampleNearbyPoint call: given a location x, a map with value $m\left(y\right)$ at y, and a kernel ρ, this returns a random point z sampled with probability proportional to $m\left(z\right)\rho \left(z\right)$. We then choose the mate as the individual nearest to that point. The approximation is examined in Appendix F.

### 9.4 Resource‐Explicit Competition: Monarchs and Milkweed

In the preceding examples, we have regulated populations through competitive interactions between individuals: either explicitly, as in Box 1, or in a space‐averaged manner, as in Box 8. Population regulation in this model is managed quite differently. This is a “resource‐explicit” model, wherein the population is extrinsically regulated by the availability of an external resource (Champer et al. 2024), as outlined in Box 10.

We simulate monarch butterflies ( *Danaus plexippus* ) and the milkweed (*Asclepias* spp.) plants on which they lay their eggs. Though adult monarchs feed on nectar from numerous types of plants, monarch caterpillars are specialists that eat only milkweed (Oberhauser et al. 2004).

BOX 10 **Resource‐explicit foraging and mortality.**

In this box, we outline the “resource‐explicit” modeling approach described more fully in Champer et al. (2024). This approach implements density‐dependent population regulation that is mediated indirectly through the availability of a resource, rather than directly through competitive interactions between individuals. A simple resource‐explicit simulation contains two species in a multispecies SLiM model: the focal species, in subpopulation p1, and a species representing the resource, in subpopulation p2, in which individuals represent patches of the modeled resource.

During the foraging phase, individuals collect resources from nearby patches. Each patch can support a certain number of individuals per time step. If a patch can support 10 individuals, but there are 100 individuals nearby at a given time step, each individual will receive 10% of the amount of resource necessary to guarantee survival (but might also forage from other nearby patches). The amount of resource that each individual collects during the foraging phase is stored in the tagF property.

1	i1.evaluate(c(p1, p2));2	for (patch in p2.individuals) {3		customers = i1.nearestNeighbors(patch, p1.individualCount, p1);4		customers.tagF = customers.tagF + inds_fed_per_patch / size(customers);5	}

After foraging, individuals die if they have not consumed enough resources. In the code example below, this occurs when individuals reach age 2.

57	at_risk_indivs = p1.subsetIndividuals(minAge=2);58	mortality_indices = runif(size(at_risk_indivs)) > at_risk_indivs.tagF;59	dead = at_risk_indivs[mortality_indices];60	species.killIndividuals(dead);

If an individual collected at least one unit of the resource, its survival to the next time step is guaranteed. Otherwise, it survives with a probability equal to the total amount it collected. See Appendix F for discussion of the relationship to the method of Box 8.

#### 9.4.1 Modeling Approach

Monarchs in the model progress through three life‐cycle stages: caterpillar, pupa, and butterfly. Mortality is modeled differently in each stage. The food resource, milkweed, is directly included in the model as a second species.

**FIGURE 11:**
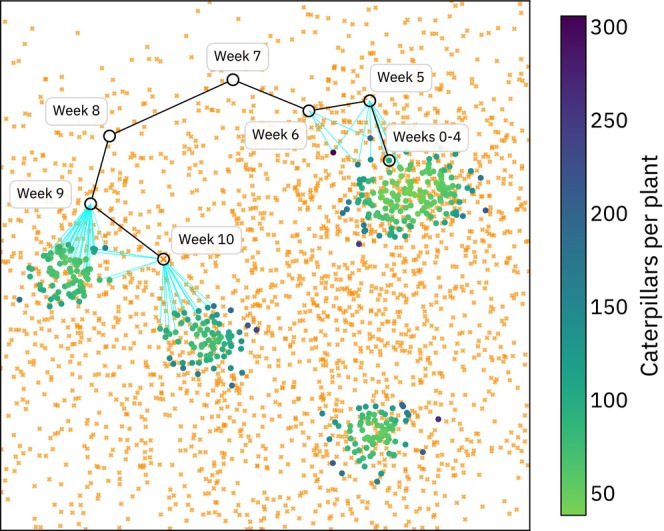
A portion of the landscape with butterflies (orange) and milkweed (green). There are about 40,000 caterpillars and 400 milkweed plants in this area. Each plant provides sufficient resources to allow an average of two caterpillars to survive to adulthood per week. A total of 2680 butterflies are present in this area. The life history of a single individual is tracked from its origin on a particular milkweed plant, with black lines depicting dispersal, and teal lines showing where this individual laid eggs.

During the first 2 weeks of their lives (time steps 0 and 1), caterpillars interact with nearby milkweed plants and accumulate resources. The amount of resources collected from a milkweed plant each week is inversely proportional to the number of competing caterpillars within a given interaction scale ($\sigma_{X}$) of that plant. This means that a plant fed upon by more caterpillars will be depleted more quickly. When individuals reach their third week, they enter the pupa phase. At this time, survival is proportional to the amount of milkweed eaten as a caterpillar. (The survival probability is calculated only once when it becomes a pupa.) Surviving pupae become butterflies during their fifth week. Butterflies disperse, reproduce, and experience mortality at an age‐dependent rate. Mortality is not density‐dependent during the butterfly stage. Figure 11 shows a snapshot of the simulation, highlighting the dispersal and reproduction of individuals across the landscape.

Reproduction also involves a resource‐explicit interaction, since monarchs only lay their eggs on milkweed. To accomplish this, a spatial interaction is evaluated between adult males and milkweed (with a much longer range than the interaction between caterpillars and milkweed); each milkweed caches a list of nearby males from that interaction. Next, a similar spatial interaction is evaluated between adult females and milkweed; females are iterated through, with each female selecting a mate from the males cached at the milkweed plants within the female's interaction range. Finally, the females randomly distribute their eggs at the plants where matings occurred.

#### 9.4.2 Observations and Extensions

In addition to reflecting the life history of monarch butterflies, the resource‐explicit modeling approach is highly performant. Each female monarch can lay several hundred eggs, very few of which go on to reach adulthood. As a result, the number of caterpillars in the model far exceeds the number of milkweed plants. Thus, regulating the size of the caterpillar population by evaluating a spatial interaction between caterpillars and plants is far more efficient than regulating the population by evaluating an interaction directly between caterpillars.

This approach also provides an intuitive way to investigate questions related to resource availability. For example, this model could probe the effects that milkweed habitat loss, caused by urbanization or climate change, could have on the viability of the local monarch population.

## 10 Discussion

The first spatial models intended to represent landscapes (as opposed to, say, two‐deme island‐mainland models) were based on partial differential equations and so did not explicitly represent individuals (Skellam 1951; Beverton and Holt 1957; Cantrell and Cosner 2004). Many others have used arrays of discrete populations (e.g., metapopulation models, Hanski 1997), or had individuals living on a regular grid (Epperson 2003). Although there are use cases for these, the main reason that individual‐based continuous‐space models are not more common may simply be convenience, both for mathematical analysis and software programming. The continuous‐space formulation we use was introduced by Pacala (1986) and Bolker and Pacala (1997), and has been used in many theoretical studies (Dieckmann and Law 1999; Snyder and Chesson 2004; Etheridge et al. 2024).

A more modern extension to matrix population models, integral projection models, more commonly incorporate density dependence (Ellner et al. 2016), and may even estimate functional forms—see for example Adler et al. (2010). Much of the remaining literature on density dependence only considers the form of $F\left(u\right)$, rather than separating out the effects of density on different life‐history components (reviewed in Caswell 2000; Eskola and Geritz 2007). There are a wide variety of methods to infer $F\left(u\right)$ from time series data (Lande et al. 2003), but statistical issues make the problem difficult (Freckleton et al. 2006). However, the “$F\left(u\right)$” thus inferred is not necessarily the same as ours—many of these methods assume no demographic stochasticity, and hence an infinite population size. In other words, the $F\left(u\right)$ that these infer is a *landscape‐scale* relationship, averaging the net effects of birth and death over thousands or millions of individuals. Our $F\left(u\right)$, on the other hand, is local, and describes how a single individual is affected by having a few more or less neighbors. These are related, but need not have the same functional forms. In particular, the behavior of $F\left(u\right)$ for very large or small values of u is usually much more important when applied to an individual, because the local density around an individual might be proportionally much larger or smaller than average, due to random fluctuations, than the total population size across a landscape. These can have a strong effect—for instance, increasing population density can have a positive effect on population growth (for at least low enough densities), a dynamic which can have interesting and important effects (Courchamp et al. 2008).

Furthermore, the effects of density dependence are often mediated (at least in part) through interactions with other species, and the nature of these interactions may depend on environmental context. These interactions are often modeled as the net result of pairwise interactions, and various methods are used to estimate these potentially numerous and environmentally dependent effects (for recent examples, see Weiss‐Lehman et al. 2022; Malyon et al. 2023). It is beyond the scope of this article to review the full range of approaches and possibilities—but note that there is no obstacle to simulation of multiple species whose interactions vary across space and/or time in SLiM (Haller and Messer 2023).

### 10.1 Discretized Continuous Space

There is not a hard distinction between “continuous” and “discrete” spatial models. However, in practice, discretized models usually only allow density‐dependent regulation to act *within* demes: in other words, vital rates of an individual can only depend on the other individuals in the same deme. This is important because the degree of demographic stochasticity depends on deme size; therefore, different discretizations may produce different amounts of stochasticity. What demographic stochasticity there is shared among all individuals in the same deme, reducing the overall variance in the simulation. The effects of these discretization artifacts are not well described (but see Battey et al. 2020). If demographic rates within each deme instead depended on nearby demes, then this would not be an issue. However, we are not aware of any simulators that make this choice.

### 10.2 Empirical Estimation of Density Dependence

Huge amounts of observational effort have gone into detailed estimates of the demography of particular species, especially those of conservation or management concern. However, such observations by necessity describe a snapshot of demographic rates in particular conditions (or, averaged over a particular range of conditions). Estimating the functional responses to density necessary to describe long‐term dynamics is much more difficult, although methodological progress has been made, for instance, by incorporating many sources of information with “integrated population models” (Zipkin et al. 2023). A great deal of ecological work has also sought to quantify the effects of density‐dependent demographic feedback. In a spatial model, this population density is usually measured locally—around the individual in question—yielding concepts such as the “competition kernel” (Bolker and Pacala 1997) or “crowding index” (Pacala and Silander 1985). Density‐dependent feedback has a key place in the theory of coexistence between species: the Janzen‐Connell hypothesis suggests that stronger intra‐ than interspecific negative density‐dependent effects could provide a mechanism for species coexistence (Janzen 1970; Connell 1971; Terborgh 2012; Hülsmann et al. 2021). A substantial number of studies across a variety of organisms (mostly plants) have quantified these effects, both within species (e.g., Weiner 1982; Silander and Pacala 1985; Specht and Arnold 2018; Spotswood et al. 2017) and between (e.g., Mack and Harper 1977; Song et al. 2021; Zaiats et al. 2021). Estimation of density‐dependent interaction strengths between many species in a community is challenging, but important for understanding community assembly (e.g., Weiss‐Lehman et al. 2022; Malyon et al. 2023). Adding to this complexity, the effects likely often depend strongly on age (Richardson et al. 2024).

Researchers in plant ecology have made the most progress toward empirical understanding of the mechanistic underpinnings of the sort of local density dependence we require. In practice there are a great many possible ways to quantify the cumulative effect of the neighbors of a single individual (Weigelt and Jolliffe 2003). Our formulation here treats all individuals equivalently, but in practice, one could include the effects of age or size. For instance, a common method in forestry modeling defines a “neighborhood competition index” for a given tree as the sum over all neighbors of their diameter (to some power) divided by distance (to another power) (Bella 1971; Daniels 1976; Canham et al. 2004). Empirical studies have estimated these kernels in a variety of situations: for instance, Teller et al. (2016) and Adler et al. (2018) use spline methods to flexibly estimate the effects of total area of nearby plants on a target plant's growth and survival.

### 10.3 Empirical Estimation of Dispersal

The concept of a dispersal distribution (as discussed in Section 4) is perhaps most well defined for plants, which (mostly) have only one opportunity to move during their lifetime, as a seed or other propagule. Seeds and pollen can be moved by gravity, wind (Nurminiemi et al. 1998), water (Murray 2012), or animals (Morales and Morán López 2022; Pons and Pausas 2007), and complex models for these have been developed and estimated from empirical data (Neubert et al. 1995; Tufto et al. 1997; Austerlitz et al. 2004; Katul et al. 2005). Of course, many animals have relatively stable locations as well, perhaps depending on the season: for instance, Paradis et al. (1998) review estimates of post‐natal and breeding dispersal in many bird species. The combination of these various processes (which often happen across many different spatial scales) has been referred to as the “total dispersal kernel” (Rogers et al. 2019), and can easily lead to “long‐tailed” distributions (Cain et al. 2000; Edwards et al. 2007) in which rare, long‐distance events are important. Dispersal is notoriously hard to estimate, as it often requires observing rare events, but has been done in a variety of organisms including kangaroo rats (French et al. 1968), mosquitoes (Estep et al. 2010), *Drosophila* (Dobzhansky and Wright 1943), *Prunus* shrubs (Robledo‐Arnuncio and García 2007), pine pollen (Robledo‐Arnuncio and Gil 2005), and butterflies (Suchan et al. 2024). Dispersal can of course also depend on density (Harman et al. 2020) (e.g., if individuals preferentially disperse out of crowded areas). Clobert et al. (2012) review many aspects of dispersal, from parameterization and estimation to implications for ecology and evolution. See also Saastamoinen et al. (2018); Edelaar and Bolnick (2012).

## 11 Conclusion

Individual‐based simulations are a powerful method for studying how demographic and population‐genetic processes operate over continuous geographic space. Modelers must design rules for how individuals in the simulation interact with others nearby and how forces such as selection operate. Individual‐based simulations are well suited to this problem since they are very flexible and can be tailored to a specific research system. However, flexibility can be both a blessing and a curse: it is easy to design a simulation with unstable population dynamics or unrealistic life‐history traits. Similarly, stochasticity can cause a population that is intended to equilibrate to instead die out. Such problems likely reveal a flaw in our understanding of the system being modeled.

Here, we provide guidance and connections to the ecological literature for researchers interested in designing stable, efficient, and interpretable spatial simulations. Interpretability is a key advantage of spatial, individual‐based simulations, since it can take substantial effort to translate the results of more abstracted models back to the domain of interest.

Realism is not a goal of our case studies, but each illustrates the degree of realism that can be obtained from SLiM without serious effort. Increasing computational efficiency and flexibility of simulation engines are bringing individual‐based simulations closer to realistic models of the ecology of specific systems. Careful implementation of ecologically realistic evolutionary models will be important to many applied fields, such as understanding and predicting how climate change affects organisms' ranges, predicting the consequences of a gene‐drive release in the wild, and rescuing species close to extinction. As the scale and specificity of *in silico* models improve, individual‐based simulations will become valuable tools beyond academic research for management professionals in conservation, management, and public health.

### 11.1 Modeling Density Dependence

Setting out to write this paper, we hoped to provide a comprehensive yet simple guide to best practices in implementing density‐dependent population regulation. Although we have provided one or two paths forward and elucidated many of the issues (see in particular Appendix B), careful empirical practitioners will soon encounter additional questions. What are some flexible and robust families of functional forms, and what aspects of these matter in practice? How can these be parameterized so that parameters naturally correspond to observable/interpretable quantities? How can these be fit to data? How should local habitat quality and density interact? At first, we imagined that the answers would be found in familiar names, and so the relationship between local density and fitness would be described as logistic, Beverton–Holt, Ricker, *et cetera*. However, we quickly found that these models were developed to describe population‐level net changes, and so they not only do not account for individual‐level stochasticity, but furthermore do not separate birth from death. This is an area of active work—see, for instance, Aoyama et al. (2022) and Adler et al. (2018) for recent good examples. Full exploration of these questions was too much for this paper.

### 11.2 Spatial Data and Niche Modeling

Several of our case studies use environmental variables to specify where on the simulated landscape organisms are most likely to live. The explosion in remote‐sensing data provides many potential data sources for modeling spatial heterogeneity. However, what is often needed in a model is a composite proxy for “suitability” that can be incorporated into local demographics. The process of predicting where a species might or does live is known as Ecological (or, Environmental) Niche Modeling (Booth et al. 1988; Peterson 2001). This can be done in a variety of ways; for instance, one might model either the potential or the realized niche and predict probabilities of occurrence or population densities (reviewed in Sillero 2011). Environmental niche models are often used to predict suitable habitat either in other locations or other time periods (Werkowska et al. 2017; Yates et al. 2018), but resulting estimates can vary widely in quality and there are a number of statistical pitfalls (Sillero and Barbosa 2021) that simulation testing could diagnose and simulation‐based inference could potentially help avoid.

While ecological niche modeling uses observational data to predict where organisms might live, a collection of landscape genetics tools tries to use genetic relatedness to predict where organisms move. The methods generate a map of “landscape resistance” that aims to describe how easily individuals move over different parts of the landscape (McRae et al. 2008). However, resistance models rely on correlating genetic distance to an abstract notion motivated by electrical circuits (reviewed in Peterson et al. 2019; Cruzan and Hendrickson 2020). They lack an underlying mechanistic model, so estimates can be problematic in practice (Cushman et al. 2013; Graves et al. 2013), and are expected to mislead in plausible situations such as biased dispersal (Lundgren and Ralph 2019). Again, simulation‐based inference provides a promising route forward (Smith et al. 2023, 2024), since it does not rely on explicit likelihoods or other mathematical descriptions.

### 11.3 Sampling

Any simulation study that wishes to make comparisons to real data needs to also consider the sampling effort that led to those data, and realistic simulation of many sampling schemes can be daunting. In practice, sampling can strongly affect results (for an example in population genetics, see Battey et al. 2020). However, it is relatively easy to assess the robustness of results to variations in the sampling scheme. Furthermore, it is often possible to “oversample” simulations. For instance, in Smith et al. (2023), many simulated datasets can be obtained from each costly spatial simulation, simply by repeating the sampling effort (however, if sufficient simulations are not done, model performance will be poor). It would be useful to develop a standard set of tools that implements various sampling schemes for simulated spatial populations.

### 11.4 The Future

Although the spatial simulations we present here incorporate many more aspects of real organisms' lives than does the Wright–Fisher model, there are many things that we have not tried to explicitly model, such as seasonal migration, herding or flocking, territoriality, foraging strategies, microhabitat variation, broadcast spawning, resource storing, pollination, predation, facilitation, and other inter‐species interactions. Any of these can be modeled in SLiM with more or less effort, and indeed many are described in the SLiM manual (Haller and Messer 2024). The decision of which aspects of biology to model is in practice made by prioritizing those aspects expected to substantially affect the question being studied. We are excited to see the wide variety of simulations that researchers develop in the future, as we explore these questions and build on each other's work.

## Author Contributions

**Elizabeth T. Chevy:** conceptualization (lead), investigation (lead), methodology (lead), visualization (lead), writing – original draft (lead), writing – review and editing (lead). **Jiseon Min:** conceptualization (lead), formal analysis (lead), investigation (lead), methodology (lead), visualization (lead), writing – original draft (lead), writing – review and editing (lead). **Victoria Caudill:** conceptualization (supporting), investigation (supporting), methodology (supporting), visualization (supporting), writing – original draft (supporting), writing – review and editing (supporting). **Samuel E. Champer:** conceptualization (supporting), investigation (supporting), methodology (supporting), visualization (supporting), writing – original draft (supporting), writing – review and editing (supporting). **Benjamin C. Haller:** conceptualization (supporting), methodology (lead), software (lead), supervision (supporting), writing – review and editing (supporting). **Clara T. Rehmann:** conceptualization (supporting), investigation (supporting), methodology (supporting), visualization (supporting), writing – original draft (supporting), writing – review and editing (supporting). **Chris C. R. Smith:** conceptualization (supporting), investigation (supporting), methodology (supporting), visualization (supporting), writing – original draft (supporting), writing – review and editing (supporting). **Silas Tittes:** conceptualization (supporting), investigation (supporting), methodology (supporting), visualization (supporting), writing – original draft (supporting), writing – review and editing (supporting). **Philipp W. Messer:** funding acquisition (equal), supervision (supporting), writing – review and editing (supporting). **Andrew D. Kern:** conceptualization (supporting), funding acquisition (equal), supervision (supporting), writing – review and editing (supporting). **Sohini Ramachandran:** conceptualization (equal), funding acquisition (equal), supervision (equal), writing – review and editing (equal). **Peter L. Ralph:** conceptualization (equal), funding acquisition (equal), investigation (equal), project administration (equal), software (equal), supervision (equal), visualization (equal), writing – original draft (equal), writing – review and editing (equal).

## Conflicts of Interest

The authors declare no conflicts of interest.

## Supporting information


Appendix S1.

## Data Availability

SLiM scripts suitable for reuse of all simulations used in this paper are at https://github.com/kr‐colab/spatial_sims_standard (for SLiM v4.2 Haller and Messer 2023). Scripts to produce the figures in this manuscript are available at https://github.com/kr‐colab/spatial_sims.
