# Correction to “Population Genetics Meets Ecology: A Guide to Individual‐Based Simulations in Continuous Landscapes”

**DOI:** 10.1002/ece3.71540

**Published:** 2025-06-05

Chevy, E.T., Min, J., Caudill, V., Champer, S.E., Haller, B.C., Rehmann, C.T., Smith, C.C.R., Tittes, S., Messer, P.W., Kern, A.D., Ramachandran, S., and Ralph, P.L. (2025), Population Genetics Meets Ecology: A Guide to Individual‐Based Simulations in Continuous Landscapes. Ecol Evol, 15: e71098.

In Figure 10 caption, the citation “Global Biodiversity Information Facility (GBIF)” should be replaced with "GBIF.org (21 December 2022) GBIF Occurrence Download https://doi.org/10.15468/dl.8pukaa", following the GBIF data citation guideline.

We apologize for this error.

