# Supplementary material for: Population Genetics Meets Ecology: A Guide to Individual‐Based Simulations in Continuous Landscapes
Source: Ecol Evol. 2025 Apr 15;15(4):e71098. doi: 10.1002/ece3.71098 (PMC11997375; doi:10.1002/ece3.71098)
Supplement: Supplementary file 1 — Appendix S1. [file ECE3-15-e71098-s001.pdf]

Appendix of “Population genetics meets ecology: a guide to individual-based simulations in continuous landscapes”

Elizabeth T. Chevy[1*], Jiseon Min[2*], Victoria Caudill[2], Samuel E. Champer[3], Benjamin C. Haller[3], Clara T. Rehmann[2], Chris C. R. Smith[2], Silas Tittes[2], Philipp W. Messer[3], Andrew D. Kern[2,4], Sohini Ramachandran[1], and Peter L. Ralph[2,5]

[*]These authors contributed equally to the paper.
[1]Center for Computational Molecular Biology, Brown University, Providence RI 02912, USA
[2]Institute of Ecology and Evolution, University of Oregon, Eugene OR 97402, USA
[3]Department of Computational Biology, Cornell University, Ithaca NY 14853, USA
[4]Department of Biology, University of Oregon, Eugene OR 97402, USA
[5]Department of Data Science, University of Oregon, Eugene OR 97402, USA

March 27, 2025

Appendix A Example SLiM script

Here is a complete SLiM script for a spatial simulation with local Beverton-Holt regulation on mortality.

```
initialize() {
  initializeSLiMModelType("nonWF");
  initializeSLiMOptions(dimensionality="xy");

    defineConstant("SD", 0.3);          // sigma_D, dispersal scale
    defineConstant("SX", 0.3);          // sigma_X, interaction scale for measuring local
    density
    defineConstant("SM", 0.3);          // sigma_M, mate choice scale
    defineConstant("K", 5);             // carrying capacity per unit area
    defineConstant("FECUN", 0.25);      // mean number of offspring per time step
    defineConstant("WIDTH", 25.0);      // width of the simulated area
    defineConstant("HEIGHT", 25.0);     // height of the simulated area
    defineConstant("RUNTIME", 200);     // total number of ticks to run the simulation for

  // Set up constants that depend on externally defined parameters
  defineConstant("RHO", FECUN / ((1 + FECUN) * K));

  // basic neutral genetics
  initializeMutationRate(1e-8);
  initializeMutationType("m1", 0.5, "f", 0.0);
  initializeGenomicElementType("g1", m1, 1.0);
  initializeGenomicElement(g1, 0, 1e8-1);
  initializeRecombinationRate(1e-8);

  // spatial interaction for local density measurement
  initializeInteractionType(1, "xy", reciprocal=T, maxDistance=3 * SX);
  i1.setInteractionFunction("n", 1, SX);

  // spatial interaction for mate choice
  initializeInteractionType(2, "xy", reciprocal=T, maxDistance=3 * SM);
  i2.setInteractionFunction("n", 1, SM);
}

1 first() {
  sim.addSubpop("p1", asInteger(K * WIDTH * HEIGHT));
```

```
  p1.setSpatialBounds(c(0, 0, WIDTH, HEIGHT));
  p1.individuals.setSpatialPosition(p1.pointUniform(p1.individualCount));
}

first() {
  // preparation for the reproduction() callback
  i2.evaluate(p1);
}

reproduction() {
  mate = i2.drawByStrength(individual, 1);
  if (mate.size())
    subpop.addCrossed(individual, mate, count=rpois(1, FECUN));
}

early() {
  // Disperse offspring
  offspring = p1.subsetIndividuals(maxAge=0);
  p1.deviatePositions(offspring, "reprising", INF, "n", SD);

  // Measure local density and use it for density regulation
  i1.evaluate(p1);
  inds = p1.individuals;
  competition = i1.localPopulationDensity(inds);
  inds.fitnessScaling = 1 / (1 + RHO * competition);
}

late() {
  if (p1.individualCount == 0) {
    catn("Population went extinct! Ending the simulation.");
    sim.simulationFinished();
  }
}

RUNTIME late() {
  catn("End of simulation (run time reached)");
  // code for output might go here
  sim.simulationFinished();
}
```

Appendix B Pitfalls

Even after carefully parameterizing a simulation to equilibrate near a given population density, it is fairly easy in practice to end up with a spatial simulation that mysteriously dies out or behaves oddly in other ways. A less dramatic annoyance is that usually the realized population size is not equal to the desired density, K, multiplied by the total area. Indeed, developing a formula for expected total population size in terms of the simulation parameters that is better than a rough first-order approximation seems extremely difficult. This section describes the root causes of these issues and ways to diagnose them. The discussion gets into the weeds, so here is a summary of what to check if density is not what you expect (details below):

1. Visualize the simulation to check for odd dynamics or spatial patterns, such as a regular array of clumps.

2. Make sure the neighborhood sizes N_X, N_M, and N_D are not too small (if in doubt, observe the effects of increasing σ_X, σ_M and/or σ_D).

3. Look at the mean density *experienced by individuals*, not the total density across the landscape. If you want instead to set the total population size, you'll need a post-hoc adjustment to K as in Box 2.

4. Make sure the *stage* you're measuring density in agrees with the theoretical calculations (*i.e.*, between birth and death or between death and birth).

5. Consider stochasticity: density varies randomly across the landscape, making the realized mean density differ from K.

Although it is natural to expect that the realized density of a simulation will be exactly the specified value of K, it is important to remember in practice that having a density different from K is not necessarily a problem: instead, it may reflect the natural biological consequences of the chosen model.

B.1 Why are there weird regular clumps?

At its most extreme, a dispersal scale much smaller than the interaction scale can lead to strange, regular arrays of clumps. (Clumps may appear for many other reasons, but here we're talking about a regular, hexagonal grid of clumps.) Examples are shown in Figure S8. Although such regular patterns formed by this mechanism rare in nature, they are easy to accidentally produce in simulation (and are one reason it is important to visualize the simulation, as in Box 2). For discussion of this strange phenomenon, see Sasaki (1997), Etheridge et al. (2024), or the "Spatial competition and spatial mate choice in a nonWF model" section of the SLiM manual (Haller and Messer, 2024). These are probably an indication that the dispersal or interaction scale are not well-chosen, but may indicate something more interesting.

B.2 Why does my simulation run so slowly?

The runtime of an individual-based simulation is at least proportional to the total number of individuals. However, it is common for the runtime of spatial simulations to grow *more* than linearly in the number of individuals, because of spatial dynamics that involve a large number of pairwise comparisons or interactions (such as spatial mate choice and spatial competition). Performance problems resulting from this can often be diagnosed by looking for *large* neighborhood sizes: if N_X (the interaction neighborhood size) or N_M (the mating neighborhood size) are large, one may encounter slow run times. Happily, there are solutions that can often be applied.

Perhaps the most obvious solutions are to directly reduce the number of pairwise interactions. One way is to shrink the neighborhood sizes of the model, by reducing σ_M and/or σ_X. However, that will often noticeably change the behavior of the model and sacrifice biological realism. Another way is to shrink the neighborhood size is to cut off the spatial kernel at a shorter distance, with little loss of exactitude; for a Gaussian spatial kernel, for example, cutting off at two standard deviations rather than three can reduce runtime with (perhaps) little change in dynamics, since interacting individuals 2–3 standard deviations from the focal individual interacted with that individual quite weakly anyway. However, this cannot cut runtime by more than about half, so for most models with large neighborhood sizes, another strategy is needed.

A second option is to use a "resource node" approach, as demonstrated in the monarchs example (Section 9.4), which effectively mediates the many possible individual-individual interactions with a smaller number of interactions between each individual and a nearby node (representing a localized amount of resources). The approach is discussed more fully in Champer et al. (2024).

A third option is to use spatial map operations to approximate the pairwise interactions more efficiently. Effectively, a map of the population's density can be computed in each tick of the model and then used to look up the density near each individual as a summary of all of the pairwise interactions it receives (Box 8). It turns out the same method can be used to efficiently pick nearby mates as well (Box 9). This option is in fact equivalent to using a regularly tiled grid of resource nodes, and can be proven to be a good approximation: see Appendix F.

Figure S1 shows that using the map-based approximation methods described in Boxes 8 and 9 makes it much easier to scale simulations to much higher neighborhood sizes. A naive implementation of pairwise interactions would result in runtimes that are quadratic in total population size, and hence totally infeasible for all but very small populations. Standard pairwise interactions in SLiM use efficient data structures (k-d trees) and a maximum distance cutoff (here, of 3σ), but still compute all pairwise interactions out to the maximum distance, and so are quadratic instead in neighborhood size (N_X or N_M), shown as solid lines in Figure S1. Models using spatial map-based methods or a regularly tiled grid of resource nodes, on the other hand, scale linearly with neighborhood size. These faster methods are approximate, but correspond closely, especially at high densities, as shown in Figure S2.

Each option has pros and cons, and may alter the behavior of the model. If the natural dynamics of the species are mediated through discrete locations (e.g., feeding or locations or mating sites), then adapting the resource node method is probably the most natural method. (In fact, it has been suggested that uncommon insects gather in discrete locations to find mates for not dissimilar reasons (Alcock, 1987).) If not, it may be more natural to use map-based methods. Finally, it may be necessary (especially for development purposes!) to simply model a smaller landscape.

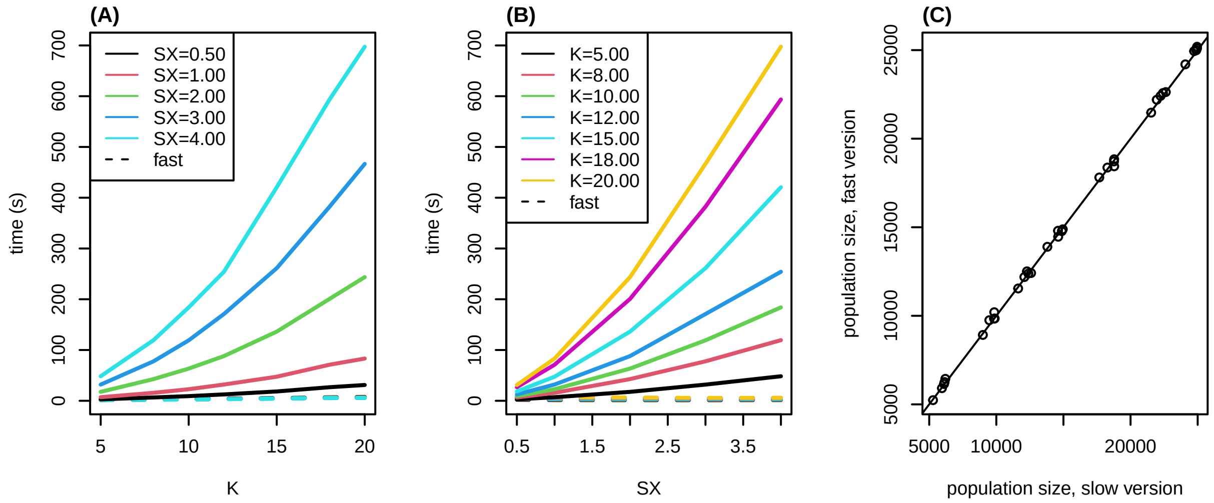

Figure S1: Runtimes for a "minimal" model with local mate choice and density-dependent Beverton–Holt feedback on mortality, plotted against **(A)** carrying capacity (K) and **(B)** interaction scale (σ_X, written `SX`). Runtimes are shown for models that do both mate choice and local density computations (solid lines) using (pairwise) interactions, and (dotted lines, all overlapping) using spatial maps, as described in Boxes 8 and 9. Also, **(C)** final population sizes after 100 time steps for the same combinations of K and σ_X shown in (A) and (B), for otherwise equivalent models that use either pairwise interactions (horizontal axis, "slow version"), or spatial map methods (vertical axis, "fast version").

B.3 Why does my simulation die out?

There are a variety of reasons why a simulation might die out (or have far fewer individuals than you expect). For instance, this can happen if σ_M, σ_X, or σ_D are too small. In all cases, "too small" can be diagnosed by looking at the relevant neighborhood sizes: for example, if $N_M = 4K\pi\sigma_M^2$ is small (less than about 1), there may be a problem related to σ_M. This problem manifests as individuals being unable to reproduce because they cannot find a mate. This is particularly likely to happen if the mating scale has been made smaller to reduce runtime (see the previous section for discussion). Solutions might be either to increase σ_M, to allow selfing, or to increase the fecundity of those individuals that do reproduce, depending upon the biology of the system being modeled. The result also depends on the mating scheme, as shown in Figure S6.

The reasons that small σ_X can be a problem are more subtle. Since σ_X determines the range over which density is computed, and each focal individual itself counts towards its local population density, then if σ_X is sufficiently small even a single isolated individual can have "local density" greater than the carrying capacity. This effect is demonstrated in Figure S7, in which the population dies out for small σ_X. The effect also appears in Figure S6, in which selfing simulations die out at low K (and hence low N_X) – since they self, they are not dying out due to small N_M. Conceptually, this happens if the simulated individuals cannot range over a large enough area to obtain sufficient resources for survival, even in the absence of competition. Since the density of a single individual calculated by equation (1) is $\rho(0)/\sigma_X^2$, then this will occur if $\rho(0)/\sigma_X^2$ is close to or greater than K, *i.e.*, roughly if $\sigma_X \leq 1/\sqrt{K}$. This could be avoided by excluding the focal

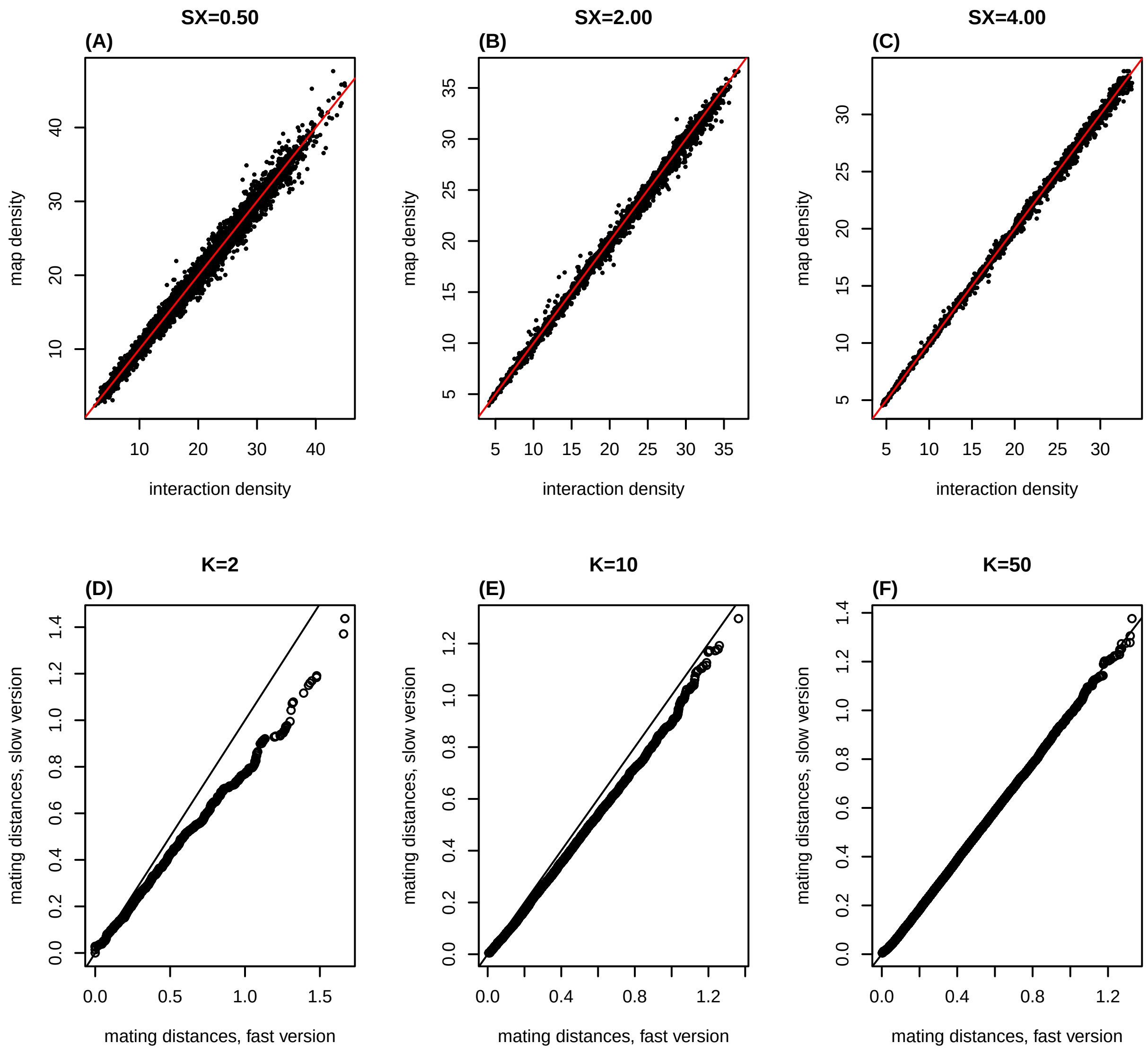

Figure S2: **(A-C)** Local density around each of 10^4 individuals, measured both using pairwise interactions (equation (1)) and interpolation on a spatial map (Box 8). Each panel shows 10^4 individuals randomly sampled from a separate simulation of the type described in Figure S1, but with K increasing linearly over 20 time steps, and different values of σ_X (labeled `SX`). **(D-F)** Q-Q plots comparing realized distributions of mating distances between simulations as above but either using individual-based mate choice (horizontal axis, "slow version") or using map-based mate choice as in Box 9 (vertical axis, "fast version"), at three values of density (K). Shown are the quantiles of the distances to the most recent mate for roughly 30,000 individuals in each simulation; at lower densities, the "fast version" tends to have slightly longer mating distances.

individual from the calculation of local density; however, this leads to the opposite problem: densities can get much larger than K. This happens because if $\sigma_X \leq 1/\sqrt{K}$, a single neighbor within scale σ_X will lead to a "local density" of more than K – but further away neighbors are unaffected. If local density increases mortality, a cartoon version of the situation is that an individual with a neighbor within distance σ_X is killed, but neighbors further away than this are ignored; as a result, the density equilibrates to around $1/\sigma_X^2$, rather than K. (The above discussion is in two dimensions; in a one-dimensional model the equilibrium

density would be around $1/\sigma_X$.)

The population can die out if σ_D (and/or σ_V) is too small for similar reasons: conceptually, if offspring do not disperse far enough from their parents and local competition is strong, then families reduce their own fitness. For a simple example, suppose that the probability that a new offspring with local density n survives is $e^{-n/K}$ and σ_D is much less than σ_X. Then, a group of m siblings form a clump of $m+1$ individuals with their parent, with density at least $(m+1)\rho(0)/\sigma_X^2$; so, the expected number of surviving offspring is smaller – at most $m\exp(-(m+1)\rho(0)/\sigma_X^2)$ – and the smaller number should be used in calculations of net reproductive output. However, if new individuals move sufficiently far (with σ_V) before local density effects, then the effect may be avoided.

The effects of changing σ_X, σ_D, and removing the focal individual from density calculations are shown in an example in Figure S9.

B.4 Why is the realized density not equal to K?

We've carefully set things up so that the equilibrium density in a neutral, spatially homogeneous simulation "should be" equal to K. If the realized density is very different (*e.g.*, zero) then the problem is probably one of the pitfalls described above. But even if N_X, N_M, and N_V are not small, realized density still often differs from K by 20% or 30%. Perhaps the simplest reason is "edge effects," but we assume the range is large enough these are unimportant (and, in practice SLiM computes local population density in such a way that local density is unaffected by edges). Another simple reason could be that density differs at different points in the life cycle – see below for more discussion of this.

First, we need to consider: *which* realized population density should we compare to K? The first answer that might spring to mind is "number of individuals divided by total area;" however, what matters for equilibrium is the density *experienced by individuals*. In other words, to see theoretical predictions playing out, we should measure local population density for each individual, and average that across individuals – that is after all the density that matters to the dynamics. This is seen in Figure S7, where mean density around individuals is shown in blue and number of individuals divided by area is shown in red, as well as in Figure S6, where mean density around individuals is shown in dotted lines and number of individuals divided by area in solid. Conceptually, if at equilibrium the simulation is very patchy (so individuals tend to be bunched up), then the mean density experienced by individuals could be much higher than the number of individuals divided by total area. In fact, the mean density around individuals is almost always be higher than the number of individuals divided by total area.

This line of reasoning leads to the second point: across individuals, local population density is a distribution, not a single value. It turns out that this stochasticity can also be important. Conceptually, the decrease in net reproduction of individuals with higher than average density may not be balanced by those individuals with lower density; how this happens depends on the shape of $F(u)$ and the shape of the distribution of densities. Below, we work through both these issues in more detail.

B.4.1 Mean density around individuals

Why is the density experienced by individuals higher than the number of individuals divided by total area? Concretely, suppose that $u_i = n(x_i)/K$ is the scaled local density for individual i; the expected change in population size is zero if $\sum_i F(u_i) = 0$. Equivalently, if U is the scaled local density for a randomly chosen individual, equilibrium occurs if $\mathbb{E}[F(U)] = 0$. Suppose instead that we look at the distribution of local densities across *space* instead of across individuals. Heuristically, suppose that we divide the landscape up into many small regions, each of area ϵ, and let v_j be the scaled density in region j (*i.e.*, $n(y_j)/K$ for some point y_j in region j, and the regions are small enough that density is constant within each). The number of individuals in region j is $\epsilon K v_j$, and so the net contribution to the next step's population size from region j is $\epsilon K v_j F(v_j)$. This equilibrium occurs if $\sum_j v_j F(v_j) = 0$. Equivalently, if we let V denote the local density around a uniformly chosen point on the landscape, then equilibrium occurs if $\mathbb{E}[VF(V)] = 0$. In fact, the relationship between U and V is that U is a *size-biased* draw from V; the relationship between the two is that $\mathbb{E}[f(U)] = \mathbb{E}[Vf(V)]/\mathbb{E}[V]$ for *any* function f. In particular, $\mathbb{E}[U] = \mathbb{E}[V^2]/\mathbb{E}[V] > \mathbb{E}[V]$ (by Jensen's inequality), and $\mathbb{E}[V]$ is just the total number of individuals divided by the total area (except for some edge effects).

Sharp-eyed readers of this and the next section will notice that we are sweeping something under the rug: when we measure density using equation (1), we do not include the focal individual. Taking this into account properly when defining V is much less clean, so for illustrative purposes we have omitted this. In fact, if individual locations are independent and uniformly distributed, then mean density around individuals (measured without the focal individual!) is equal to the number of individuals divided by total area. Nonetheless, we think the calculations are informative.

B.4.2 Stochasticity

Now we can immediately see how stochasticity interacts with nonlinearity in density dependence to increase or decrease equilibrium density. First suppose that F is convex and decreasing, *i.e.*, $F''(u) > 0$ and $F'(u) \leq 0$ for all u. Then by Jensen's inequality, $\mathbb{E}[F(U)] > F(\mathbb{E}[U])$, and since at equilibrium, $\mathbb{E}[F(U)] = 0$, we have that $F(\mathbb{E}[U]) < 0$. Since we've assumed that $F(1) = 0$ and F is decreasing, this implies that $\mathbb{E}[U] > 1$, *i.e.*, a convex F *increases* the equilibrium mean density experienced by individuals above K. By the same argument if F is concave, $\mathbb{E}[U] < 1$.

We can make the same argument for the total density, V: if $G(v) = vF(v)$ is convex, then $\mathbb{E}[V] > 1$, while if G is concave then $\mathbb{E}[V] < 1$. This at first seems odd: if $F(u)$ is convex and $uF(u)$ is concave, then the mean density experienced by individuals is *higher* than K, while the total density is *lower* than K. However, this is perfectly possible, and in fact seen in Figure S7.

A Taylor expansion lets us estimate more precisely the deviation of equilibrium size from K. Taylor expanding $F(u)$ about $u = 1$, we get that

$$\begin{aligned} 0 = \mathbb{E}[F(U)] &\approx \mathbb{E}\left[F(1) + (U-1)F'(1) + \frac{1}{2}(U-1)^2 F''(1)\right] \\ &= (\mathbb{E}[U]-1)F'(1) + \frac{1}{2}\operatorname{var}[U]F''(1) + \frac{1}{2}(\mathbb{E}[U]-1)^2 F''(1). \end{aligned}$$

If the deviation is small (*i.e.*, $|\mathbb{E}[U]-1| \ll |F'(U)/F''(U)|$, $(\mathbb{E}[U]-1)^2 \ll \operatorname{var}[U]$), the second order term is negligible. Thus, we can write:

$$\mathbb{E}[U] \approx 1 - \frac{1}{2}\operatorname{var}[U]\frac{F''(1)}{F'(1)}, \qquad \text{(S1)}$$

when $\frac{1}{2}\operatorname{var}[U] \ll \left(\frac{F'(1)}{F''(1)}\right)^2$. Since $F'(1)$ is negative, agrees with the argument above. Figure S10 shows that this prediction bears out well in practice (in two nonspatial models), as long as the population does not go extinct.

Similarly,

$$\begin{aligned} 0 = \mathbb{E}[F(U)] &= \mathbb{E}[VF(V)] \\ &\approx \mathbb{E}\left[1F(1) + (V-1)(F(1) + 1F'(1)) + \frac{1}{2}(V-1)^2(2F'(1) + 1F''(1))\right] \\ &\approx (\mathbb{E}[V]-1)F'(1) + \frac{1}{2}\operatorname{var}[V](2F'(1) + F''(1)), \end{aligned}$$

and hence

$$\mathbb{E}[V] \approx 1 - \operatorname{var}[V]\left(1 + \frac{F''(1)}{2F'(1)}\right),$$

which is smaller than $\mathbb{E}[U]$, and further away from 1, except in extreme circumstances.

What determines the strength of stochasticity? Since this has to do with random variation in "experienced" density across the landscape, stochasticity goes down as interaction neighborhood size N_X increases. In other words, if σ_X is larger, then we measure density averaging over larger areas, which is therefore less variable. As in equation (S1), stochasticity affects equilibrium by a factor proportional to $\operatorname{var}[U]$. If Y is the number of individuals within distance σ_X of a random individual, then $\mathbb{E}[Y]$ is around N_X, and if noise

is Poisson then also $\text{var}[Y] \approx N_X$. Since U is obtained by dividing $n(x)$ (from equation (1)) by K, and $n(x)$ is roughly $Y/\pi\sigma_X^2$, we expect $\text{var}[U]$ to be of order $\text{var}[Y]/(\pi\sigma_X^2 K)^2 = N_X/N_X^2 = 1/N_X$. So, we expect the deviation of realized density from K to be of order $1/N_X$. Again, this is seen in Figure S7: the form of density dependence has a convex $F(u)$, and so for smaller values of σ_X, the value of $\mathbb{E}[U]$ (blue line) is above K. Conversely, the function $uF(u)$ is concave, and so $\mathbb{E}[V]$ (red line) is increasing, but is well below K for other reasons.

The difference between the mean density around individuals ($\mathbb{E}[U]$) and the mean density by area ($\mathbb{E}[V]$, or total population size divided by total area) is well-known: the ratio $\mathbb{E}[U]/\mathbb{E}[V]$ is a scale-dependent measure of clustering known as *mean crowding* (Lloyd, 1967) that increases the more clustered individuals are on the scale used to measure local density.

B.4.3 Density measurement timing

The other thing to consider is: *when* is density being measured? In each time step there are some births and some deaths; we follow SLiM in taking births first in the time step, but since the two alternate, this choice seems arbitrary. However, having density effects or movement occur in one or the other stage can affect the model (e.g., Taylor, 2010). Following SLiM as we do, the most common "population size" is at the end of the time step, *i.e.*, after deaths (or equivalently, before births). However, population size may also be measured (and used!) between births and deaths.

Concretely, suppose that N_t is the population size at the start of time step t, and N_t^+ is the population size after births in time step t. Above, we did calculations like this: if the mean fecundity in time step t is f_t, and the mean probability of survival is $1 - \mu_t$, then

$$\mathbb{E}[N_t^+] = N_t(1 + f_t) \tag{S2}$$

$$\mathbb{E}[N_{t+1}] = N_t^+(1 - \mu_t) \tag{S3}$$

$$= N_t(1 + f_t)(1 - \mu_t). \tag{S4}$$

Which density is used to determine f_t and μ_t? Naturally, f_t can't depend on the number of births, so it will use N_t (*i.e.*, the density at the start of the time step). However, should the density dependence for μ_t use N_t or N_t^+? If the newborn individuals contribute to density, then μ_t should depend on N_t^+, *i.e.*, density computed using the offspring as well. However, this will be larger than the density at the start of the time step by a factor of $1 + f$, and so the equation for the local net per capita reproductive rate analogous to equation (2) is

$$F^+(u) = (1 + f(u))(1 - \mu(u(1 + f(u)))) - 1. \tag{S5}$$

As before, equilibrium would be around density n_* solving $F^+(n_*/K) = 0$, and so to arrange as before for the equilibrium density to be around K we'd like to set up the functional forms so that $F^+(1) = 0$. One way to do this is to start with functional forms for $f(u)$ and $\mu(u)$ so that $F(u) = (1+f(u))(1-\mu(u))-1$ satisfies $F(1) = 0$ (*i.e.*, formulated for measuring density between death and birth), and then define the survival probability to be $1 - \mu^+(u) = 1 - \mu(u/(1 + f(1)))$. Then $F^+(u)$ defined using $\mu^+(u)$ satisfies $F^+(1)$. This is the approach taken by Battey et al. (2020). Another approach is to compute a density *map*, as in Box 8, at the start of each time step, and use that map to determine density for juveniles as well.

Appendix C Parameterization of density dependence

If one wants to use a familiar phenomenological model as the basis for density dependence, there are several popular choices for the function form of $F(u)$. Here, we summarize these, parameterized so that $F(1) = 0$ (and so will have an equilibrium near $n_* = K$):

- $F(n) \propto 1 - n$, (Discrete-logistic model)
- $F(n) \propto \frac{1+a}{1+an} - 1$, (Beverton–Holt model)
- $F(n) \propto \frac{(1+a)^c}{(1+an)^c} - 1$, (Hassell model)

- $F(n) \propto e^{r(1-n)} - 1$. (Ricker model)

(Here, $\propto$ indicates that each can be scaled by a constant, reflecting an overall time scaling.)

Using each one still requires a number of choices. Next, we work through in more detail the steps involved in arranging birth and death rates so that the net birth rate, F, has a particular functional form, and give a number of examples that help to show the issues involved. Suppose here that each time step has birth followed by death; death applies in the same way to individuals just born as those previously alive; the mean fecundity of an individual with local density N is $f(N/K)$; and the probability of death of an individual with local density N is $\mu(N/K)$.

Roughly, the net change in population size when at scaled density $u = N/K$ is $F(u) = f(u)(1-\mu(u)) - \mu(u)$. However, this is not not necessarily right, since it depends when the densities are measured: if the density for mortality is measured after birth, then the density passed to μ will be different than that passed to f. So, we'll always define $F(u)$ to be the mean per-capita change in population size across one step when starting at scaled population size u. However, which point in the time step (*i.e.*, after birth and before death or vice-versa) is the reference point will depend on the situation. Our goal is to figure out how to arrive at a given functional form for F in various scenarios.

Fecundity regulation: If death probability is constant: $\mu(u) = \mu_0$, then taking u to be the scaled population density before birth, simply

$$F(u) = f(u)(1-\mu_0) - \mu_0,$$

and so

$$f(u) = \frac{F(u) + \mu_0}{1 - \mu_0}.$$

Since $0 \leq f < \infty$, for this to make sense we need $\mu_0 > 0$ and $F \geq -\mu_0$.

Beverton–Holt fecundity regulation: With $F(u) = \alpha((1+a)/(1+an) - 1)$, this is

$$f(u) = \frac{1}{(1-\mu_0)} \left(\alpha \frac{(1+a)}{(1+au)} + (\mu_0 - \alpha) \right), \tag{S6}$$

and we need $\alpha \leq \mu_0$.

Ricker fecundity regulation: With $F(u) = \alpha(e^{r(1-u)} - 1)$, this is

$$f(u) = \frac{1}{1-\mu_0} \left(\alpha e^{r(1-u)} + (\mu_0 - \alpha) \right),$$

and we again need $\alpha \leq \mu_0$.

Mortality regulation: Suppose instead that fecundity is constant: $f(u) = f_0$, and that we measure density for mortality *after* birth (so it includes the new births). Then

$$F(u) = f_0 - \mu(u)(1 + f_0),$$

and so the survival probability is

$$1 - \mu(u) = \frac{1 + F(u)}{1 + f_0}.$$

Since we must have $0 \leq \mu \leq 1$, we require that $-1 \leq F \leq f_0$. Note that in this model (regardless of the form of F), the death probability at the stationary point ($u = 1$, since $F(1) = 0$) is $\mu(1) = 1/(1 + f_0)$; and so the mean lifetime is $1 + f_0$. Also note that this produces an equilibrium density of K *before death* (as opposed to before birth, in the previous models); the density will be lower after death.

Beverton–Holt mortality regulation: With $F(u) = \alpha((1+a)/(1+au) - 1)$, this is

$$1 - \mu(u) = \frac{1}{(1+f_0)} \left(\alpha \frac{(1+a)}{(1+au)} + (1-\alpha) \right), \tag{S7}$$

and we need $\alpha a \leq f_0$.

Ricker mortality regulation: With $F(u) = \alpha(e^{r(1-u)} - 1)$, this is

$$1 - \mu(u) = \frac{1}{1+f_0} \left(\alpha e^{r(1-u)} + (1-\alpha) \right),$$

and we need $\alpha(e^r - 1) \leq f_0$.

Mortality and fecundity: Suppose now we'd like both mortality and fecundity to change with density. So that there's only one density, let's say again that is measured after birth and before death. If the density at this time is N, then the mean number of individuals in the next time step is

$$N(1 - \mu(N/K))(1 + f(N/K)),$$

so that the mean net change is

$$F(u) = (1 - \mu(u))(1 + f(u)) - 1.$$

Given a desired functional form for $F(u)$ and $\mu(u)$ we would then define

$$f(u) = \frac{F(u) + 1}{1 - \mu(u)} - 1.$$

Note that for fecundity to remain finite, we need $\mu(u)$ to be bounded away from zero. On the other hand, if we have $f(u)$ then

$$1 - \mu(u) = \frac{F(u) + 1}{1 + f(u)}.$$

Mixed Beverton–Holt: Suppose we set $f(u) = f_0/(1+bu)$ and would like $F(u) = \alpha((1+a)/(1+au) - 1)$ for some constants b and α. Then, we would set

$$1 - \mu(u) = \frac{\alpha(1+a)/(1+au) + (1-\alpha)}{f_0/(1+bu) + 1}, \tag{S8}$$

and we need $\alpha a < f_0$.

Mixed Ricker: Now suppose that we set $1 - \mu(u) = (1-\mu_0)e^{-su} + \mu_\infty(1 - e^{-su})$ and would like $F(u) = \alpha(e^{r(1-u)} - 1)$. (The extra parameters are not unnecessary complications: we will need $\alpha < 1$ and $\mu_\infty > 0$ for the following to work out.) Then, we would set

$$f(u) = \frac{\alpha e^{r(1-u)} + (1-\alpha)}{(1-\mu_0)e^{-su} + \mu_\infty(1 - e^{-su})} - 1,$$

and we need $s \leq r$ for this to remain positive. This has $f(0) = (\alpha(e^r - 1) + 1)/(1 - \mu_0) - 1$, which is positive if $r > 0$ (already a requirement). Also, $f(\infty) = (1-\alpha)/\mu_\infty - 1$, so we also need $1 - \alpha > \mu_\infty$.

Hassell with mortality regulation Let's set up the Hassell, which is $F(u) = b((1+a)/(1+au))^c - 1)$. Note that $F(0) = b((1+a)^c - 1)$, $F(\infty) = -b$, and $F'(1) = cab(1+a)^c$. Setting fecundity to be constant at f_0 and plugging in to the expression for mortality regulation above,

$$\mu(0) = \frac{f_0 - b((1+a)^c - 1)}{1+f_0}$$

and

$$\mu(\infty) = \frac{f_0 + b}{1+f_0}.$$

So, if we'd like to fix $\mu(0) = \mu_0$ and $\mu_\infty = \mu(\infty)$, then these determine a and b:

$$\begin{aligned} b &= \mu_\infty(1+f_0) - f_0 \\ a &= \left(\frac{f_0 - \mu_0(1+f_0)}{\mu_\infty(1+f_0) - f_0} + 1\right)^{1/c} - 1. \end{aligned}$$

This leaves us with a parameterization in terms of the mean fecundity, f_0, the death rate at low density, μ_0, the death rate at high density, μ_∞, and the exponent c that controls how steep the curve is between. (We could reparameterize c so we have a parameter that is exactly $F'(1)$, but this is less compelling.)

Ricker parameterization with fecundity regulation If $\mu(u) = \mu_0$ and we want $F(u) = C(e^{r(1-u)} - 1)$, then

$$f(u) = \alpha e^{-ru} + \beta, \tag{S9}$$

where letting $\alpha = Ce^r/(1-\mu_0)$ and $\beta = (\mu_0 - C)/(1-\mu_0)$. So, $f(0) = \alpha + \beta$ and $f(\infty) = \beta$. If we set $C = \mu_0$ then this is $\beta = 0$ and $\alpha = \mu_0 e^r/(1-\mu_0)$, and so $f(u) = \mu_0 e^{-r(u-1)}/(1-\mu_0)$.

Regulation by juvenile mortality Now suppose that adult death rate and fecundity are constant (so, $f(u) = f$ and $\mu(u) = \mu$), but that the probability of survival of *juveniles* is density-dependent. (Perhaps density dependence only affects the species during seedling recruitment.) So, if we call $r(u)$ the probability of survival in the first year at scaled density u, the net per capita reproduction function is $F(u) = r(u)f - \mu$. To make this proportional to the Beverton–Holt form, we can set

$$r(u) = \frac{\mu(1+a)}{f(1+au)}, \tag{S10}$$

which results in $F(u) = \mu((1+a)/(1+au) - 1)$ – *i.e.*, the Beverton–Holt form, scaled by μ.

Density regulation of both juvenile and adult mortality Now suppose that the probability of survival of juveniles (*i.e.*, to their first year) is $r(n/K)$, not $1 - \mu(n/K)$. In this case,

$$F(u) = r(u)f(u) - \mu(u),$$

and so if we fix $f(u) = f$ then we have, for instance,

$$fr(u) = F(u) + \mu(u).$$

Suppose we want $F(u) = (1+a)/(1+au) - 1$ and $\mu(u) = bu/(1+bu)$; then we would have

$$\begin{aligned} fr(u) &= \frac{(1+a)}{(1+au)} + \frac{bu}{(1+bu)} - 1 \\ &= \frac{(1+a)}{(1+au)} - \frac{1}{(1+bu)} \\ &= \frac{a}{(1+au)} + \frac{(b-a)u}{(1+au)(1+bu)} \end{aligned}$$

With this definition, $r(0) = a/f$ and $r(\infty) = (b-a)/((b+a)f)$, so we need $a \leq \max(b, f)$ (and some other conditions). Note that $r(\infty) > r(0)$ (*i.e.*, *increasing* recruitment with density) if $b - a > a(a+b)$: for instance, take $f = 1$ and $a = 1/4$ and $b = 3/4$.

Appendix D Parameterization and sampling for dispersal kernels

When sampling a new random vector for displacement or dispersal, it is simplest to think in Cartesian coordinates: to draw the displacement as (X, Y) where X and Y are independent draws from some distribution. This works well if X and Y are Gaussian, but plotting the resulting bivariate distribution quickly shows oddities: dispersers will tend to fall on either around the axes or around the diagonals, depending on the distribution chosen. In fact, displacements in orthogonal directions are independent *only* for the Gaussian distribution. To obtain a rotationally symmetric dispersal kernel, it helps to think in polar/spherical coordinates. This also brings up an issue of terminology: what do we call a given rotationally symmetric bivariate distribution? Natural choices are to name it after the shape of either (a) the profile, X, or (b) the distance, $R = \sqrt{X^2 + Y^2}$. These agree only in one dimension. We work through some examples below to make the underlying issues clear.

Gaussian (and Rayleigh) Imagine displacements along the x and y axes are sampled from the same Normal distribution with zero mean and variance of σ^2. The displacement in x, y then has the multivariate normal density

$$p(x, y) = \frac{1}{2\pi\sigma^2} \exp\left(-\frac{x^2 + y^2}{2\sigma^2}\right).$$

In polar coordinates,

$$p(r, \theta) = \frac{1}{2\pi\sigma^2} \exp\left(-\frac{r^2}{2\sigma^2}\right),$$

i.e., the density only depends on r, and so the kernel is radially symmetric.

To find the distribution of R, we can find the cumulative distribution, $\mathbb{P}(r < R)$, and differentiate. The CDF can be found by integrating $p(x, y)$ over rings of circumference $2\pi r$ and infinitesimal width of dr:

$$\mathbb{P}\{R < s\} = 2\pi \int_0^s p(x, y) r dr, \tag{S11}$$

$$= \int_0^s \frac{r}{\sigma^2} \exp\left(-\frac{r^2}{2\sigma^2}\right) dr. \tag{S12}$$

Thus, the PDF of R is $p(r) = \frac{r}{\sigma^2} \exp\left(-\frac{r^2}{2\sigma^2}\right)$, which is a Rayleigh distribution. In summary,

$$X, Y \sim N(0, \sigma^2) \tag{S13}$$

is equivalent to

$$\theta \sim \text{Uniform}(0, 2\pi), R \sim \text{Rayleigh}(\sigma) \tag{S14}$$

By the same argument, in three dimensions the angular part would be uniformly distributed on the sphere, and R has density proportional to $r^2 \exp(r^2/2\sigma^2)$.

Student's t We can do something similar with the Student's t distribution, but it will be clear that we need to be careful with generalizing it to a higher dimension.

First, what is the Student's t distribution? In one dimension, it is the distribution with density

$$p(x) = \frac{\Gamma\left(\frac{\nu+1}{2}\right)}{\sqrt{\nu\pi\sigma^2}\Gamma\left(\frac{\nu}{2}\right)} \left(1 + \frac{x^2}{\nu\sigma^2}\right)^{-(\nu+1)/2}, \tag{S15}$$

where ν is the "degrees of freedom" and σ is a scale parameter. If $\nu = 1$, it is a Cauchy distribution, and if $\nu \to \infty$, the distribution converges to a standard Normal distribution. The Student's t distribution is a heavy tailed distribution: all moments of order ν or higher are not defined.

Now suppose we want to "use the Student's t" in two dimensions. A **wrong** way to do this is to sample x and y independently from the same t distribution. If you do that, the joint distribution $p(x,y)$ is $p(x)p(y)$ due to independence, and it looks like

$$p(x,y) = \frac{\Gamma\left(\frac{\nu+1}{2}\right)^2}{\nu\pi\sigma^2\Gamma\left(\frac{\nu}{2}\right)^2}\left(\left(1+\frac{x^2}{\nu\sigma^2}\right)\left(1+\frac{y^2}{\nu\sigma^2}\right)\right)^{-(\nu+1)/2}. \tag{S16}$$

If we plug in $x = r\cos\theta$ and $y = r\sin\theta$ as we did before with the Normal distribution, we realize that $p(x,y)$ depend on both r and θ! In other words, the distribution of R depends on the angle, θ: long distances will be more common in some directions than others.

It turns out that although there are many ways to generalize the t distribution to more than one dimension, there is not a single standard way (Kotz and Nadarajah, 2004). Here are three possibilities for how we might choose a radially symmetric bivariate kernel $p(x,y)$:

1. The distribution of the distance, R, is Student's t.
2. The distribution of the distance along an arbitrary axis, X, is Student's t.
3. The shape of the kernel taken along a line through the origin is the Student's t density.

These conditions are, equivalently, that (1) $p(r,\theta) \propto r^{-1}(1+r^2)^{-(\nu+1)/2}$ (where $p(r,\theta)$ is $p(x,y)$ is radial coordinates); (2) $\int p(x,y)dy \propto (1+x^2)^{-(\nu+1)/2}$; and (3) $p(x,0) \propto (1+x^2)^{-(\nu+1)/2}$. If we chose the first option, then we'd be compelled to call the bivariate Gaussian the "bivariate Rayleigh" distribution, so for consistency with the Gaussian, we'll discard that option. Although option (2) is perhaps more elegant, figuring out what the actual density is for an arbitrary kernel shape is more involved, so we have chosen to go with option (3) (both here and in SLiM).

So: to make a radially symmetric Student's t kernel, we'd like a bivariate kernel $p(x,y)$ proportional to $p(\sqrt{x^2+y^2})$, where the second $p()$ is the univariate Student's t. With the normalization factor, for $\nu > 1$ this is

$$p(x,y) = \frac{(\nu-1)}{4\pi\nu\sigma^2}\left(1+\frac{x^2+y^2}{\nu\sigma^2}\right)^{-(\nu+1)/2}. \tag{S17}$$

The density of R, the displacement distance, is equal to $2\pi r p(r,0)$.

$$p_R(r) \propto \frac{r}{\sigma}\left(1+\frac{r^2}{\nu\sigma^2}\right)^{-(\nu+1)/2}. \tag{S18}$$

Note that if $\nu = 1$ (*i.e.*, the Cauchy distribution), the integral $\int_0^\infty p_R(r)dr$ does not converge. This means $\nu = 1$ gives an invalid probability distribution, and so in two dimensional space, the number of degrees of freedom should be greater than 1. (More generally, in d dimensions we'll need $\nu > d-1$.)

General kernels To generalize this, begin with a univariate probability distribution with density $f(x)$. Then, we define another distribution for r by $p_R(r) \propto r^{d-1}f(r)$ if in d dimensions. This is equivalent in two dimensions to defining a joint distribution of x,y by $p(x,y) = f(\sqrt{x^2+y^2})$. To sample a random variable (X,Y) in two dimension, we sample R from $p_R(r)$, sample θ from the uniform distribution on $(0,2\pi)$, and set $X = R\cos\theta$ and $Y = R\sin\theta$. (More generally, we would choose the radial component to be uniform on the d-sphere; the easiest way to do that is to let $(X_1,\ldots,X_d) = R(Z_1,\ldots,Z_d)/\sqrt{Z_1^2+\cdots+Z_d^2}$, where $Z_1,\ldots,Z_d$ are independent standard Normal.) This is how the function `pointDeviated()` in SLiM returns a displaced locations from various rotationally symmetric kernel.

D.1 Sampling from kernels with a covariance matrix

The multivariate Normal is not necessarily rotationally symmetric: it allows a general covariance matrix; if the covariance matrix is not a multiple of the identity, then the contours of the density are ellipses, not circles. How might we introduce covariance matrices to multivariate dispersal? The most convenient way to

do this is to use *scale mixtures* of Normals, *i.e.*, just multiply a given multivariate Normal distribution (with some covariance matrix) by a random scaling, choosing the distribution of the random scaling appropriately. For instance, if we'd like a fat-tailed dispersal kernel whose level sets are ellipses rotated by an angle θ counterclockwise, we can write:

```
U = rgamma(1, NU/2, NU/2);
  dxprime = rnorm(1, 0, SD_X) / sqrt(U);
  dyprime = rnorm(1, 0, SD_Y) / sqrt(U);
pos = individual.spatialPosition + c(dxprime * cos(THETA) - dyprime * sin(THETA),
          dxprime * sin(THETA) + dyprime * cos(THETA));
if (p1.pointInBounds(pos)) {
  offspring = subpop.addCrossed(individual, mate);
  offspring.setSpatialPosition(pos);
}
```

Note that we use `pointInBounds()` to check boundary condition, and so the boundary is absorbing. Here we've generated a *scale mixture of Normals*, by dividing our (correlated) multivariate Gaussian by $\sqrt{U}$, where U is Gamma($\nu/2$,$\nu/2$) distributed. This is in fact another (and arguably better) common definition of the "multivariate Student's t" (Kotz and Nadarajah, 2004).

Appendix E Additional methodological details for case studies

E.1 Temporal change: pikas

For computational efficiency, we focus on a 266 km^2 region of Rocky Mountain National Park (RMNP) in Colorado (40.40363°N to 40.53856°N; 105.7326°W to 105.5977°W). A map in WGS84 projection was obtained using the `elevatr` package in R.

By simulating a restricted area of the species range, we could overestimate the probability of extinction by missing larger spatial scale population dynamics. In addition, the resulting genetic variation will certainly be affected by modeling a smaller, narrowly distributed population.

To regulate the population we at first aim for a uniform density of 250 individuals per km^2 throughout the habitat. This value is informed by the number of pika scat piles observed by Erb et al. (2014) in sites in the Rocky Mountains. For the expected lifetime we use 3.25 years, as reported by Smith (1974). Thus, we set $K = 250$ and fecundity equal to $\frac{1}{3.25}$.

We did not find a published value for competitive interaction distance for pikas, nor for mating distance. Therefore, we decided to use the same value for the spatial scales of competition, parent-offspring dispersal, and mating (*i.e.*, $\sigma_I = \sigma_D = \sigma_M$). Our assumption will introduce error if the competitive interaction scale in pikas is different than the dispersal scale, or if the mating scale varies from the other two values, which are both likely to be true. There is no adult movement in the pika model.

This simplifying assumption might be accurate, as pikas are territorial (Smith, 1974). To choose a value for σ_X (and the other, shared interaction scales) we use as a starting point the value of 300 m from Smith (1974) which was the maximum reported distance traveled by juveniles. Assuming that parent-offspring dispersal is Gaussian distributed in each dimension, we calculated a σ_X such that three standard deviations from the mean, Euclidean distance is $300 = \sqrt{\frac{\pi}{2}}\sigma_X + 3\sqrt{(2 - \frac{\pi}{2})}\sigma_X$ (using formulas for mean and standard deviation of the Rayleigh distribution).

E.2 Complex life cycles: mosquitoes

In our model of mosquitoes, we assume that only the juveniles' viability is affected by local density, while adult population size is regulated only through a constant mortality. Due to this detail, if we want to control the juvenile density to match carrying capacity, we need to modify the density control function (such as Beverton–Holt model) to reflect the life cycle.

To start our derivation, let's say density of juveniles with age i is a_i with $i = 1, ..., m-1$ where m is maturation age. Let's also define density of adults as a_m. The adults have a fixed survival probability, $1 - \mu_a$. We use a variation of Beverton–Holt model where survival probability of juvenile population is a local population density factor, $\frac{1}{1+\rho u}$, multiplied by the baseline survival probability $1 - \mu_j$, where μ_j is the

baseline mortality of juveniles. (here u is population density of juveniles, *i.e.*, $\sum_{i=1}^{m-1} a_i$). Our goal is to find ρ.

Then we get a system of m equations for $\mathbf{a}$ (left-hand-side is the a in the next time step, but there is no time dependence by definition of equilibrium.):

$$a_1 = \frac{a_m FEC/2 \cdot (1-\mu_j)}{1+\rho(\sum_{i=1}^{m-1} a_i + FEC/2 \cdot a_m)} \tag{S19}$$

$$a_k = \frac{a_{k-1}(1-\mu_j)}{1+\rho(\sum_{i=1}^{m-1} a_i + FEC/2 \cdot a_m)} \tag{S20}$$

$$a_m = \frac{a_{m-1}(1-\mu_j)}{1+\rho(\sum_{i=1}^{m-1} a_i + FEC/2 \cdot a_m)} + a_m(1-\mu_a), \tag{S21}$$

where $k = 2, ..., m-1$. Notice that we have a factor of 2 for fecundity, FEC, because only female adults (assumed to be half of total adult population) produces FEC new individuals. ρ controls where the equilibrium density is for juveniles, and it is an unknown for now. We want ρ to make $\sum a_{i=1}^{m-1} = K$, where K is carrying capacity of larvae.

Due to recursive relation between a_i's, we can simplify the system of $m+1$ equations for $m+1$ unknowns (a_i's and ρ) to two equations with two unknowns, a_m and ρ.

To make it a little clear, let's define,

$$r \equiv \frac{1-\mu_j}{1+\rho(\sum_{i=1}^{m-1} a_i + FEC/2 \cdot a_m)} = \frac{1-\mu_j}{1+\rho(K + FEC/2 \cdot a_m)} \tag{S22}$$

Now we start from the first equation of a_1,

$$a_1 = a_m FEC/2 \cdot r \tag{S23}$$

and plug it into the next one to find a_2.

$$a_2 = a_m FEC/2 \cdot r^2 \tag{S24}$$

and keep going to a_{m-1}. We see that $a_i = a_m FEC/2 \cdot r^m$, a nice geometric series. (This makes sense because we tend to see exponential distributed age-structure in simulations.) This is nice because the sum can be simplified very nicely

$$\sum_{i=1}^{m-1} a_i = a_m \cdot FEC/2 \cdot r \cdot \frac{1-r^{m-1}}{1-r} = K. \tag{S25}$$

This is one of two equations we will need. The second one comes from plugging in a_{m-1} to the original equation for a_m.

$$a_m = a_m(1-\mu_a) + a_m FEC/2 \cdot r^m. \tag{S26}$$

Dividing both sides by a_m, and rearranging terms, we get

$$r = \left(\frac{\mu_a}{FEC/2}\right)^{1/m}. \tag{S27}$$

Plugging this into the first equation, we get a_m! And we can find ρ by plugging it into the equation we used to define r. Finally, we get a_m and ρ as a function of a_m that we have in our simulation model:

$$a_m = K \frac{1-\left(\frac{\mu_a}{FEC/2}\right)^{1/m}}{1-\left(\frac{\mu_a}{FEC/2}\right)^{1-1/m}} \cdot \mu_a^{-1/m}(FEC/2)^{1-1/m} \tag{S28}$$

$$\rho = \frac{\frac{(1-\mu_j)(FEC/2)^{1/m}}{\mu_a^{1/m}} - 1}{K + a_m \cdot FEC/2} \tag{S29}$$

In Figure S11 we compare the expected adults to juveniles ratio from the equation above to the simulated value. Even though we didn't consider other factors like spatial aspects (finding mates, migrations, heterogeneity of the river map) and seasonal fluctuation, the simulated ratio stay pretty close to theoretical expectation. In addition, I also plot the average local population density that juveniles measure around themselves through an interaction kernel with width $SX = 20.0$ in Figure S12. The density closely follows the rain factor which is a sinusoidal function added everywhere on the map to set the baseline carrying capacity, except for when the rain factor is close to zero. During the "dry season," we see increase of density because we programmed the adult females to disperse offspring to the locations where carrying capacity is high within the maximum dispersal distance.

E.3 Continental-scale systems: cane toads

Note that there has already been considerable effort to simulate the Australian cane toad invasion (Kearney et al., 2008), including simulations that incorporate genetic information to infer many biological parameters (Estoup et al., 2010).

Toads were simulated to have a juvenile state of 1 year. Individuals that were at least one year old were allowed to disperse once each year over their entire lifetime. Toads were randomly assigned to be male or female. Offspring initial locations were set to their mother's locations.

Previous studies have shown that the spread rate and distribution of cane toads has been influenced by environmental heterogeneity (Urban et al., 2008). We initially used a homogeneous landscape to model the cane toad invasion, which resulted in glaring discrepancies between the simulated and observed distributions. In efforts to increase the likeness of simulated distributions to the observed data, we incorporated environmental heterogeneity across space.

The kernels controlling competition and mate choice were kept as Gaussians.

The pipeline for downloading the complete data is available (`https://github.com/kr-colab/spatial_sims/blob/main/silas/range_expansion/pipelines/get_data.smk`), and is described here briefly. We downloaded cane toad occurrence data was from the Global Biodiversity Information Facility (`https://doi.org/10.15468/dl.8pukaa`), bioclimatic variables from WorldClim (`https://www.worldclim.org/data/bioclim.html` (Fick and Hijmans, 2017)), and a shapefile for the geographic perimeter of Australia from the Australian Bureau of Statistics (`https://www.abs.gov.au/`). We converted the locations from latitude and longitude values to km using using GeoPandas (Jordahl et al., 2020) (see notebook/vignette for further details), and set the origin of the map to the earliest location in the occurrence data.

Extensions Our approach could be extended to use multiple environmental maps along with a multivariate fitness function over each environmental dimension.

If desired, the simulations could be extended to include the ability to use and compare genetic information between observed and simulated data. SLiM's ability to model explicit genomes could also allow for more complexity and realism, such as a heritable component for dispersal ability to model assortative mating for dispersal ability, as well as gene surfing, where deleterious alleles are maintained at the range edge (Miller et al., 2020; Shine et al., 2021). Additional complexity could be incorporated into the life history traits as well. For example, cannibalism has been observed in cane toads (DeVore et al., 2021). This and other modifications could be made related to changes age related competition, fecundity, mortality, and establishment.

E.4 Resource competition: monarchs

The milkweed is only minimally simulated within this model. A number of patch centroids are randomly spread across an area of the model, and a number of milkweed plants are scattered around each centroid according to a Gaussian distribution. During each year of the model, after the monarchs have migrated south for the winter, the locations of the patch centroids and plants are re-randomized.

Appendix F Map-based approximations to density

In this section, we provide a formal argument showing that the approximation scheme of Box 8 or of a uniform tiling of resource nodes converges to the local density for a fine enough grid (for an empirical demonstration of this convergence, see Champer et al. (2024)). Conceptually, this works because the approximation effectively computes density as if all points were at the node of the region they are in. Since all the regions are small, this does not change things much.

Suppose that the positions of individuals on the landscape are recorded as a collection of points $\{x_i\}_{i=1}^N$. It will be helpful for notation to represent the state of the population as a point measure, $\Lambda = \sum_i \delta_{x_i}$. For simplicity in this section, suppose that distances are measured in units of the interaction scale, i.e., $\sigma_X = 1$. The density we would like to compute from equation (1) is then

$$n(x) = \sum_i \rho(x - x_i),$$

the convolution of Λ with the kernel ρ. Now suppose that we placed a discrete set of nodes on the landscape at locations $\{y_j\}$, and for each j let A_j denote the portion of space that is closer to y_j than to any other node. In other words, $\{A_j\}$ is the Voronoi tesselation associated with $\{y_j\}$; and suppose we assign the boundaries between regions in some sensible way. Suppose that the diameters of all A_j are less than ϵ; we will show that using these nodes we can approximate $n(x)$ well to within an error that is proportional ϵ – so, finer meshes of nodes will make better approximations.

Suppose that we're evaluating density at the location of node y_j. The approximation outlined in Box 8 seeks to approximate $n(y_j)$ by

$$\hat{n}(y_j) = \sum_k \rho(y_j - y_k)\Lambda(A_k),$$

where $\Lambda(A_k)$ is the number of individuals within the region A_k, which we may write as $\Lambda(A_k) = \sum_i 1_{A_k}(x_i)$. So, we can write

$$\begin{aligned} n(y_j) - \hat{n}(y_j) &= \sum_i \left(\rho(y_j - x_i) - \sum_k \rho(y_j - y_k) 1_{A_k}(x_i) \right) \\ &= \sum_k \sum_{i : x_i \in A_k} \left(\rho(y_j - x_i) - \rho(y_j - y_k) \right). \end{aligned} \tag{S30}$$

Now note that by the intermediate value theorem and the fundamental theorem of calculus,

$$\rho(y_j - x) - \rho(y_j - y_k) = (x - y_k)\rho'(y_j - y_k + \alpha(y_k - x))$$

for some $0 \leq \alpha \leq 1$. Now, $\rho(x) \to 0$ as $x \to \infty$, so for any δ we may pick R so that

$$\int_{|x - y_j| > R - \epsilon} \rho'(x) dx < 1/N.$$

Write $N_R = \#\{i : |x_i - y_j| \leq R\}$ for the number of points closer than R. Furthermore, suppose the derivative of ρ is bounded above by C, *i.e.*, $\|\rho'(x)\| \leq C$. If $|x - y_k| \leq \epsilon$, then

$$|\rho(y_j - x) - \rho(y_j - y_k)| \leq \epsilon C.$$

Since for $x_i \in A_k$, by definition $|x_i - y_k| \leq \epsilon$, plugging this into equation (S30), and splitting the sum into regions with nodes further than R away and from y_j and not,

$$|n(y_j) - \hat{n}(y_j)| \leq \epsilon \left(1 + C N_R\right).$$

Since C and N_R are fixed, this goes to zero as $\epsilon \to 0$. This shows that $n(y_j) \approx \hat{n}(y_j)$; since $\hat{n}(x)$ is defined for arbitrary x by interpolation, $n(x) \approx \hat{n}(x)$ as well.

Supplementary figures

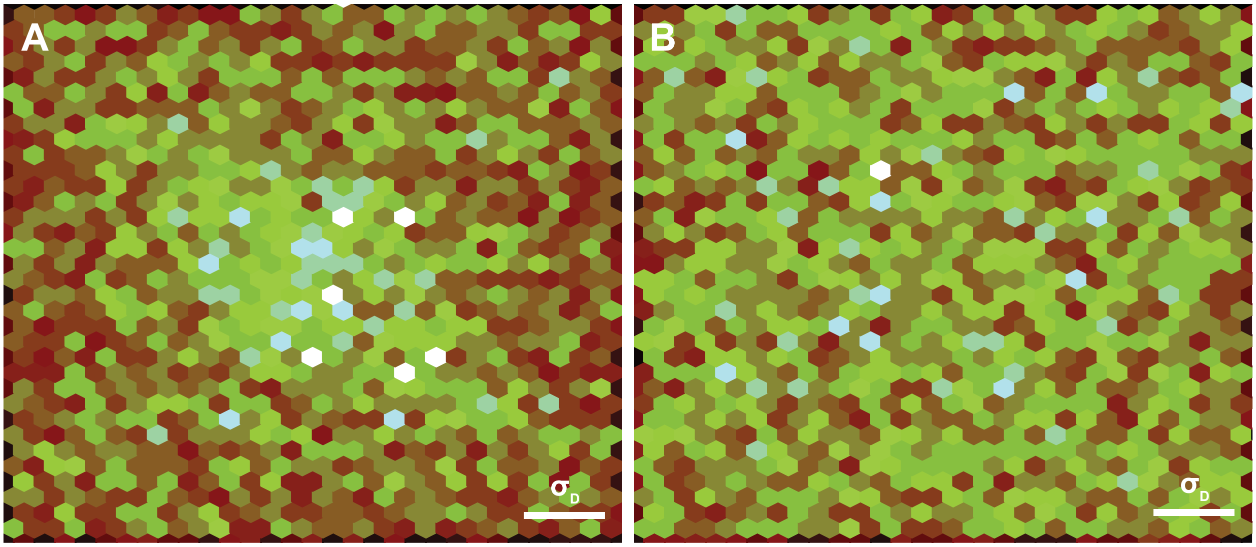

Figure S3: Fatter-tailed dispersal reduces clumping. Fry plots illustrate the distance between each pair of individuals at a given tick. The dense region of points near the origin in the Gaussian panel indicates many pairs of individuals are separated by small distances. Points shown are pairwise distances ≤ 1 from an arbitrary time step from one replicate. Here, $\sigma_D = 0.3$. **(A)** Gaussian dispersal kernel. **(B)** Student's t dispersal kernel. Figure 3CD quantify clumping visualized here.

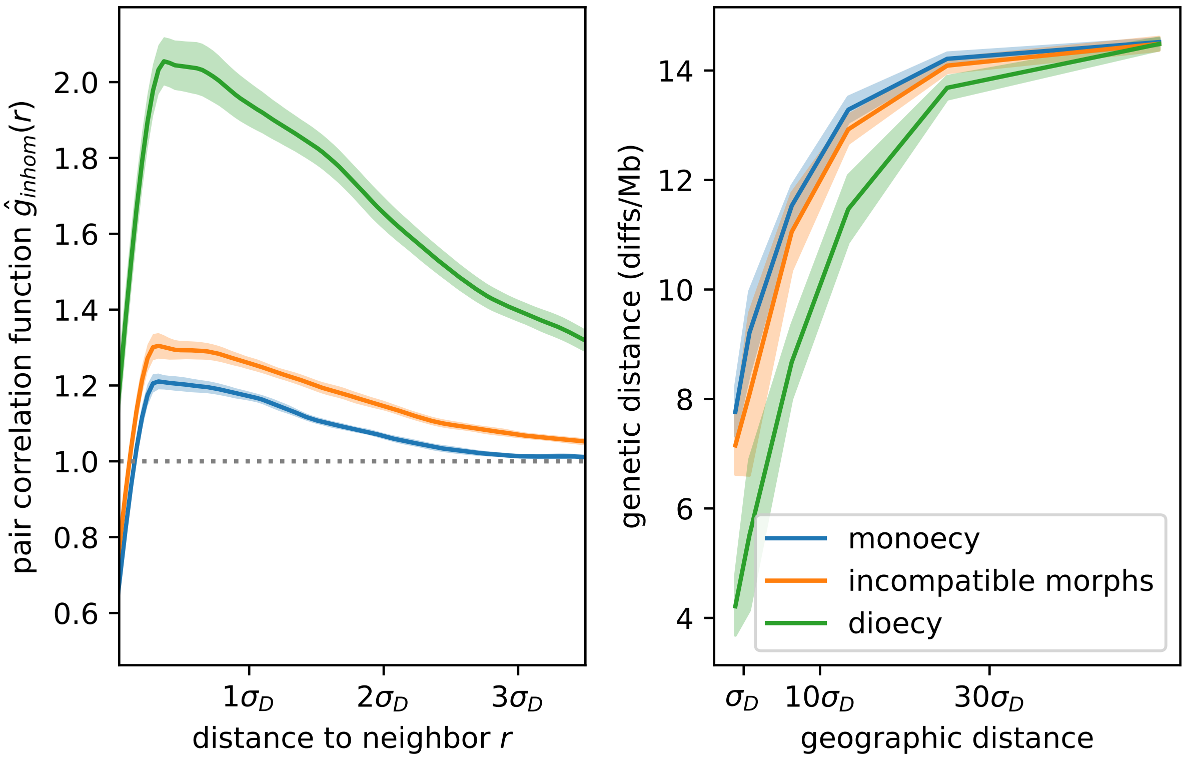

Figure S4: Mating type affects magnitude and spatial scale of clumping. **(left)** Pair correlation functions show density of pairs of individuals found a particular distance apart, relative to distances expected under a Poisson process (1.0; grey dotted line). Curves show the average across 50 independent time steps. Mating types are as described in Section 5. **(right)** Mate limitation increases genetic isolation-by-distance. Plots show mean genetic distance between pairs of individuals at increasing geographic distance, averaged across ten independent replicates. Figure 3AB visualize clumping quantified here for monoecious and dioecious scenarios, respectively.

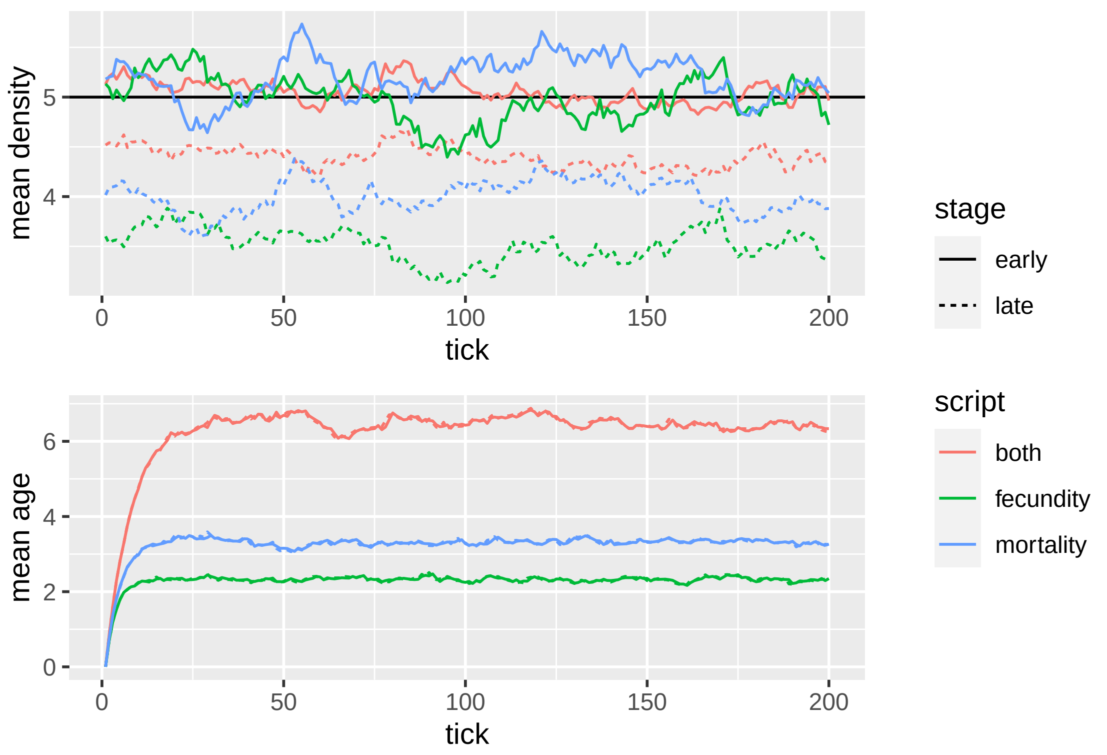

Figure S5: Traces from spatial simulations of the three "Beverton–Holt" models shown in Figure 1. **(Top)** Average local density for all individuals, measured both between birth and death ("early") and between death and birth ("late"), and **(bottom)** mean ages across 200 time steps. Horizontal line shows the value of $K = 5$; local population density determines population regulation between birth and death for all models. Other parameters: $\sigma_D = \sigma_X = 1.2$, and range was a 25×25 square area.

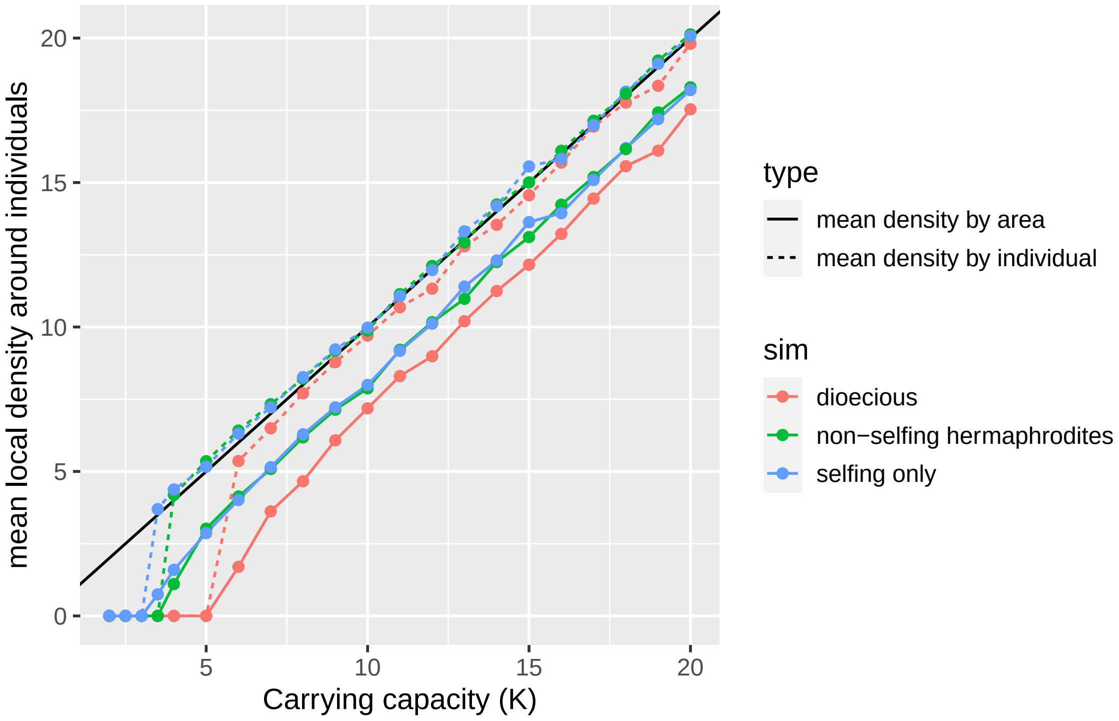

Figure S6: Average realized population density at equilibrium of three different models, plotted against the "carrying capacity" parameter, K. Each model uses Beverton–Holt regulation of mortality with the same parameters ($\sigma_D = \sigma_I = \sigma_M = 0.3$). and differ only in that "*dioecious*" individuals are one of two sexes: only females reproduce and only if they mate with a male; "*non-selfing hermaphrodites*" also must mate with another individual, but all individuals can reproduce; and "*selfing*" individuals can all reproduce and have no need for mating. Furthermore, mean fecundity is $f = 0.5$ for the dioecious simulations and $f = 0.25$ for the others. Dotted lines show mean local density experienced by individuals; solid lines show total population size divided by total area. All simulations die out at low density, but those requiring mating die out at higher K: in each case, when N_M is around 1.6.

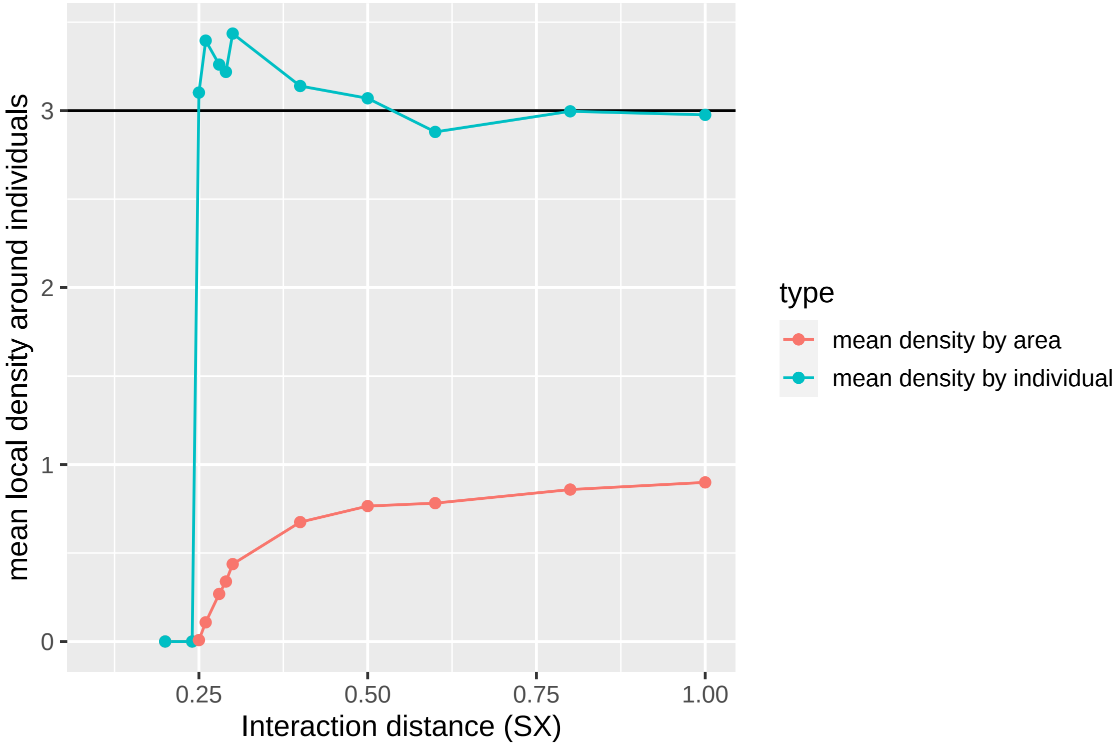

Figure S7: Mean density at equilbrium in simulations with different values of σ_X (`SX` in the figure). The blue line shows mean density around individuals (*i.e.*, the average of local density calculated with equation (1) for all individuals), the red line shows total population size divided by area (a 25 $\times$ 25 box), and the horizontal line is at $K = 3$. The simulation uses Beverton–Holt control of mortality with $\sigma_D = 0.5$, reproduction entirely through selfing, and no adult movement. The population dies out if σ_X is below 0.25, while for $0.25 \leq \sigma_X \leq 0.5$, total density (*i.e.*, density averaged by area) increases, while mean density experienced by individuals is larger than K and decreases.

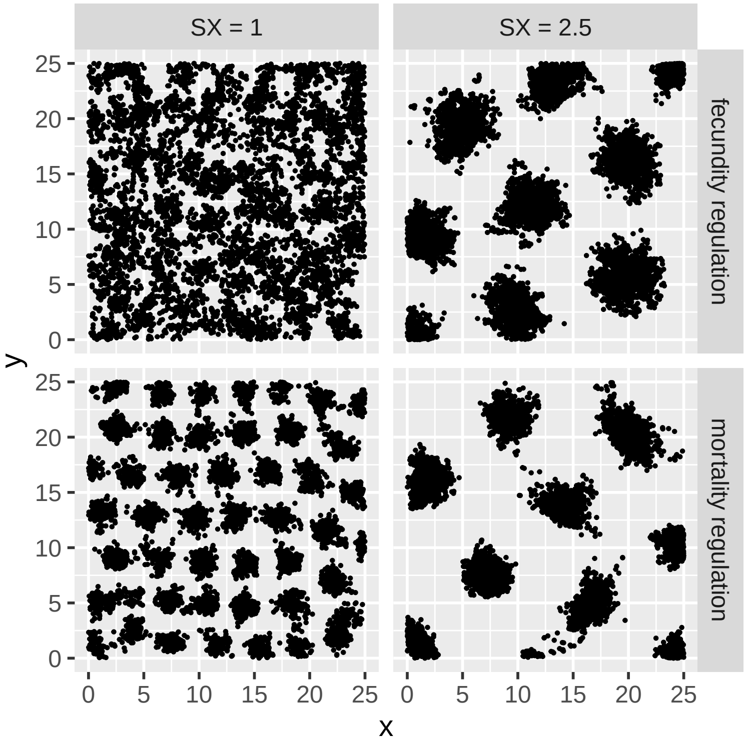

Figure S8: Examples of simulations exhibiting clumping, using $\sigma_D = 0.2$, two different values of σ_X (labeled SX), and two different types of density-dependent feedback (either mortality or fecundity regulation, as in Figure S5).

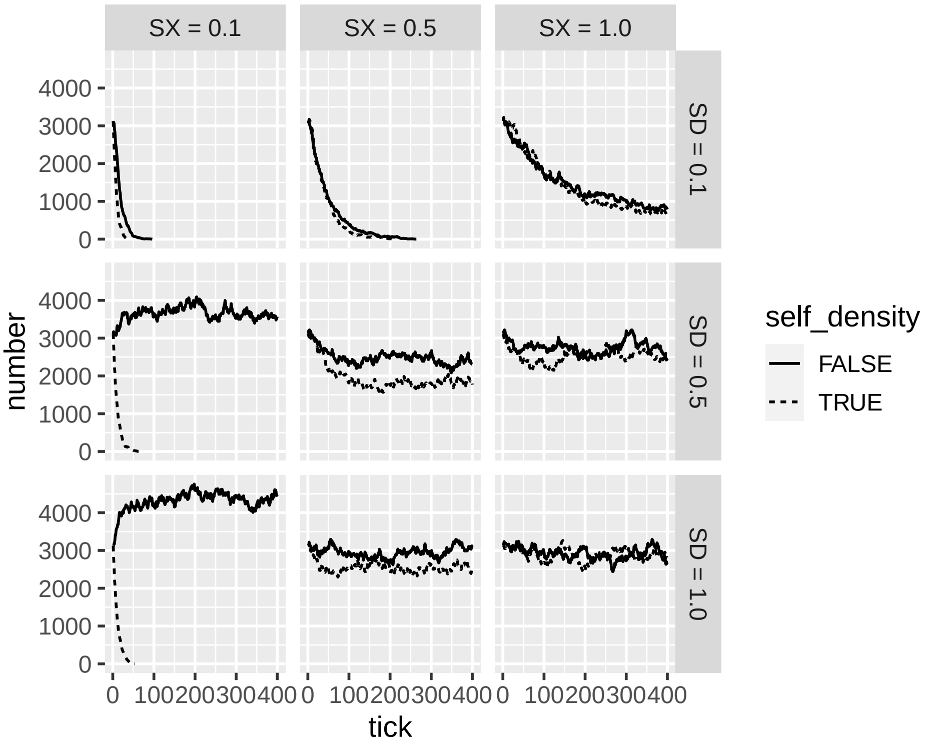

Figure S9: Population sizes through time for simulations with various values of dispersal scale (σ_D, here `SD`) and interaction scale (σ_X, here `SX`), with or without inclusion of the focal individual in local density calculations. Each simulation had Beverton–Holt density-dependent feedback on mortality (as in Figure S5), and was run with $K = 5$ on a 25×25 square area, and were started with $5 \times 25 = 3125$ individuals (so, lines that are roughly flat are fluctuating around a total density of $K = 5$). Solid lines compute "local density" for control of mortality of each individual using equation (1), while dotted lines do the same except excluding the focal individual.

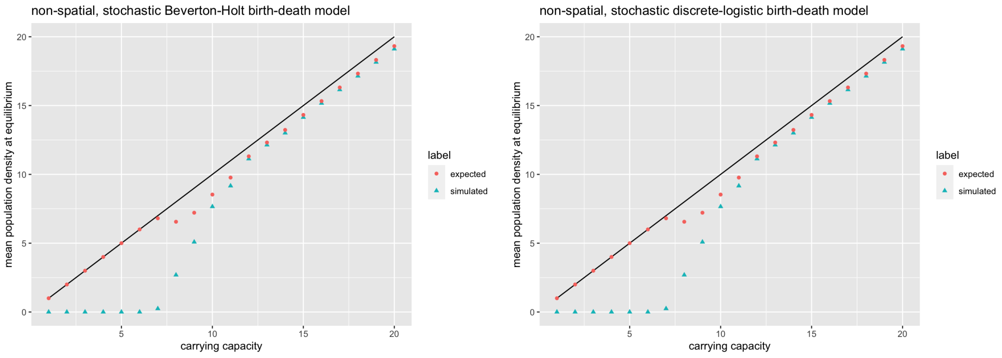

Figure S10: Expected population density obtained using equation (S1) and actual average population density in nonspatial models with **(left)** Beverton–Holt regulation of mortality for which "expected" is $K - \frac{Var[U]}{(f+1)K}$, and **(left)** discrete logistic regulation of mortality, for which "expected" is $K - \frac{Var[Y]}{K}$. Nonspatial simulations were run in R. Solid line is where density is equal to carrying capacity, which is true for deterministic model.

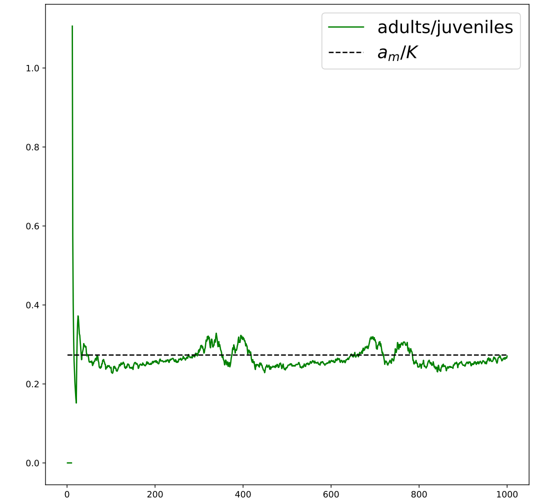

Figure S11: Ratio between adult and juvenile mosquito counts.

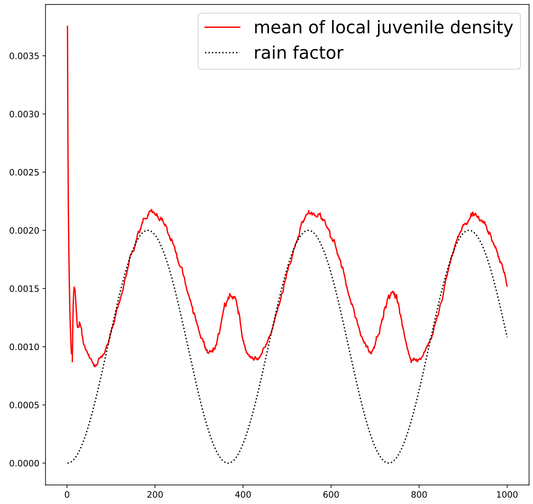

Figure S12: Local population density of juvenile mosquitoes plotted against rain factor, which is minimum carrying capacity at each time point.

References

John Alcock. Leks and hilltopping in insects. *Journal of Natural History*, 21(2):319–328, 1987. doi: 10.1080/00222938700771041. URL `https://doi.org/10.1080/00222938700771041`.

C J Battey, Peter L Ralph, and Andrew D Kern. Space is the place: Effects of continuous spatial structure on analysis of population genetic data. *Genetics*, 215(1):193–214, 05 2020. ISSN 1943-2631. doi: 10.1534/genetics.120.303143. URL `https://doi.org/10.1534/genetics.120.303143`.

Samuel E. Champer, Bryan Chae, Benjamin C. Haller, Jackson Champer, and Philipp W. Messer. Resource-explicit interactions in spatial population models. *bioRxiv*, 2024. doi: 10.1101/2024.01.13.575512. URL `https://www.biorxiv.org/content/early/2024/01/15/2024.01.13.575512`.

Jayna L DeVore, Michael R Crossland, Richard Shine, and Simon Ducatez. The evolution of targeted cannibalism and cannibal-induced defenses in invasive populations of cane toads. *Proceedings of the National Academy of Sciences*, 118(35):e2100765118, 2021.

Liesl P Erb, Chris Ray, and Robert Guralnick. Determinants of pika population density vs. occupancy in the Southern Rocky Mountains. *Ecological Applications*, 24(3):429–435, 2014.

Arnaud Estoup, Stuart JE Baird, Nicolas Ray, Mathias Currat, Jean-Marie Cornuet, Filipe Santos, Mark A Beaumont, and Laurent Excoffier. Combining genetic, historical and geographical data to reconstruct the dynamics of bioinvasions: application to the cane toad *Bufo marinus*. *Molecular Ecology Resources*, 10 (5):886–901, 2010.

Alison M. Etheridge, Thomas G. Kurtz, Ian Letter, Peter L. Ralph, and Terence Tsui Ho Lung. Looking forwards and backwards: Dynamics and genealogies of locally regulated populations. *Electronic Journal of Probability*, 29(none):1 – 85, 2024. doi: 10.1214/24-EJP1075. URL `https://doi.org/10.1214/24-EJP1075`.

Stephen E Fick and Robert J Hijmans. WorldClim 2: new 1-km spatial resolution climate surfaces for global land areas. *International journal of climatology*, 37(12):4302–4315, 2017.

Benjamin C. Haller and Philipp W. Messer. *SLiM: An Evolutionary Simulation Framework (the SLiM manual)*, 2024. URL `https://messerlab.org/SLiM`.

Kelsey Jordahl, Joris Van den Bossche, Martin Fleischmann, Jacob Wasserman, James McBride, Jeffrey Gerard, Jeff Tratner, Matthew Perry, Adrian Garcia Badaracco, Carson Farmer, Geir Arne Hjelle, Alan D. Snow, Micah Cochran, Sean Gillies, Lucas Culbertson, Matt Bartos, Nick Eubank, maxalbert, Aleksey Bilogur, Sergio Rey, Christopher Ren, Dani Arribas-Bel, Leah Wasser, Levi John Wolf, Martin Journois, Joshua Wilson, Adam Greenhall, Chris Holdgraf, Filipe, and François Leblanc. geopandas/geopandas: v0.8.1, July 2020. URL `https://doi.org/10.5281/zenodo.3946761`.

Michael Kearney, Ben L Phillips, Christopher R Tracy, Keith A Christian, Gregory Betts, and Warren P Porter. Modelling species distributions without using species distributions: the cane toad in australia under current and future climates. *Ecography*, 31(4):423–434, 2008.

Samuel Kotz and Saralees Nadarajah. *Multivariate t-Distributions and Their Applications*. Cambridge University Press, Cambridge, 2004. ISBN 9780521826549. doi: DOI:10.1017/CBO9780511550683. URL `https://www.cambridge.org/core/books/multivariate-tdistributions-and-their-applications/463E067E32C230D3205563EDD195DA2D`.

Monte Lloyd. 'mean crowding'. *Journal of Animal Ecology*, 36(1):1–30, 1967. ISSN 00218790, 13652656. URL `http://www.jstor.org/stable/3012`.

Tom EX Miller, Amy L Angert, Carissa D Brown, Julie A Lee-Yaw, Mark Lewis, Frithjof Lutscher, Nathan G Marculis, Brett A Melbourne, Allison K Shaw, Marianna Szűcs, et al. Eco-evolutionary dynamics of range expansion. *Ecology*, 101(10):e03139, 2020.

Akira Sasaki. Clumped distribution by neighbourhood competition. *Journal of Theoretical Biology*, 186(4): 415–430, June 1997. doi: 10.1006/jtbi.1996.0370. URL `https://doi.org/10.1006%2Fjtbi.1996.0370`.

Richard Shine, Ross A Alford, Ryan Blennerhasset, Gregory P Brown, Jayna L DeVore, Simon Ducatez, Patrick Finnerty, Matthew Greenlees, Shannon W Kaiser, Samantha McCann, et al. Increased rates of dispersal of free-ranging cane toads (*Rhinella marina*) during their global invasion. *Scientific Reports*, 11 (1):23574, 2021.

Andrew T Smith. The distribution and dispersal of pikas: consequences of insular population structure. *Ecology*, 55(5):1112–1119, 1974.

P. Taylor. Birth–death symmetry in the evolution of a social trait. *Journal of Evolutionary Biology*, 23(12): 2569–2578, 12 2010. ISSN 1010-061X. doi: 10.1111/j.1420-9101.2010.02122.x. URL `https://doi.org/10.1111/j.1420-9101.2010.02122.x`.

Mark C Urban, Ben L Phillips, David K Skelly, and Richard Shine. A toad more traveled: the heterogeneous invasion dynamics of cane toads in australia. *The American Naturalist*, 171(3):E134–E148, 2008.
